# Growth and mineralogy in dental plates of the holocephalan *Harriotta raleighana* (Chondrichthyes): novel dentine and conserved patterning combine to create a unique chondrichthyan dentition

**DOI:** 10.1186/s40851-019-0125-3

**Published:** 2019-03-12

**Authors:** Moya Meredith Smith, Charlie Underwood, Tomasz Goral, Christopher Healy, Zerina Johanson

**Affiliations:** 1 Centre for Craniofacial and Regenerative Biology, Faculty of Dentistry, Oral and Craniofacial Sciences, King’s College London, London, SE1 9RT, UK; 2 Department of Earth Sciences, Natural History Museum London, London, SW7 5BD, UK; 3 Department of Earth and Planetary Sciences, Birkbeck, University of London, London, UK; 4 Current address: Center of New Technologies, University of Warsaw, Warsaw, Poland

**Keywords:** Dental plates, Osteodentine, Hypermineralized dentine, Whitlockite, Dental evolution, Holocephali

## Abstract

**Abstract:**

The dentition in extant holocephalans (Chondrichthyes) comprises three pairs of continuously growing dental plates, rather than the separate teeth characterizing elasmobranchs. We investigated how different types of dentine in these plates, including hypermineralized dentine, are arranged, and how this is renewed aborally, in adult and juvenile dentitions of *Harriotta raleighana* (Rhinochimeridae). Individual plates were analysed using x-ray computed tomography (μCT), scanning electron microscopy (SEM) in back scattered mode with energy dispersive X-ray (EDX) analysis, and optical microscopy on hard tissue sections.

**Results:**

*Harriotta* dental plates are made entirely of dentine tissue, mostly as trabecular dentine, bone itself being absent. Hypermineralized dentine forms in restricted ovoid and tritor spaces within trabecular dentine, inside a shell of outer and inner dentine layers. Trabecular dentine is ubiquitous but changes to sclerotic osteodentine near the oral surface by increasing density, remaining less mineralized than the hypermineralized dentine. All structures are renewed aborally, within a vascular dental pulp, a tissue suggested to be a source of stem cells for tissue renewal. Ca density profiles and concentrations of Mg, P, and Ca ions reveal extreme differences in the level and type of mineralization. Early mineralization in ovoids and tritors has very high levels of Mg, then a sudden increase in mineralization to a high total mineral content, whereas there is gradual change in trabecular dentine, remaining at a low level.

Hypermineralized dentine fills the prepatterned ovoid, rod and tritor spaces, early at the aboral surface within the trabecular dentine. Deposition of the hypermineralized dentine (HD, proposed as new specific name, whitlockin replacing pleromin) is from surfaces that are lined with large specialized odontoblasts, (whitloblasts, instead of pleromoblasts) within cell body spaces connecting with extensive, ramifying tubules. Early mineralization occurs amongst this maze of tubules that penetrate far into the dentine, expanding into a mass of saccules and membranous bodies, dominating in the absence of other organic matrix. This early stage has hydroxyapatite, also significantly rich in Mg, initiated as a poorly crystalline phase. In the hypermineralized dentine, formation occurs as clusters of variably shaped crystals, arising from a sudden phase transition.

**Conclusions:**

In the hypermineralized dentine, high MgO + CaO + P_2_O_5_ suggests that almost pure Mg containing tricalciumphosphate (MgTCP: (ß-Ca_3_(PO_4_)_2_) (whitlockite) is present, with little or no hydroxyapatite. Serial replacement of tritors and ovoids is suggested to occur within the dental plate, probably representing a relic of patterning, as classically found in elasmobranch dentitions.

## Background

Within the Chondrichthyes, the Holocephali (chimaeroids) form the sister group to the Elasmobranchii (sharks, skates, rays [1]). The elasmobranch dentition is increasingly well understood, including how teeth are continually produced from residual stem cells in a permanent dental lamina that patterns all replacement teeth, generated along the jaws [2, 3]. The dentition in crown group holocephalans is not developed from separate teeth, but instead includes six dental plates, as a statodont (non-shedding) dentition, each growing continuously from pulpal tissue. Here, dentine forms both a trabecular tissue and a hypermineralized tritoral tissue with different wear rates on the biting (oral) surface. The distribution of this hypermineralized tissue varies between taxa; in the holocephalan family Rhinochimaeridae, hypermineralized dentine is restricted to lingual tritors and separate labial ‘strings of beads’ [1, 4, 5]. Studies of the chimaeroid *Chimaera monstrosa* demonstrated that the hypermineralized tissue includes the magnesium-containing mineral whitlockite [6], unknown as a dominant mineral in elasmobranch teeth, or any other vertebrate skeletal structure.

**Fig. 1:**
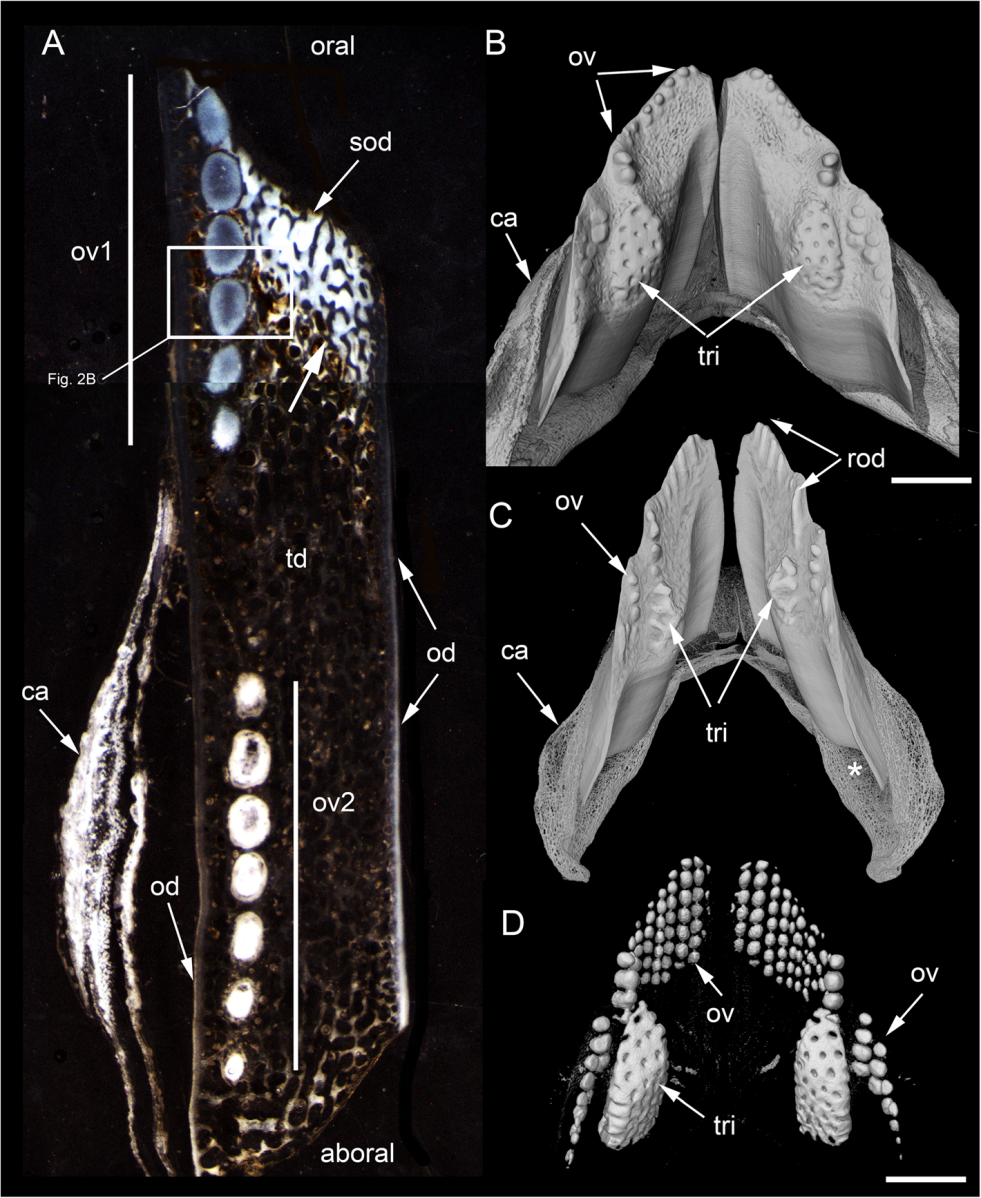
*Harriotta raleighana* (Rhinochimeridae; Holocephali; Chondrichthyes). Morphology and microstructure of adult dental plate in lower jaw. **a** adult, vertical section, left labial and right lingual. **c** juvenile, **b**, **d** adult, μCT renders of paired plates, dorsal view. **a** rostralmost section through ovoids only (no tritor), incident reflected illumination reveals different tissues relative to their degree of mineralization complimentary with the density differences in μCT images (**b–d**). Grey, translucent, most mineralized HD of in set of five ovoids (ov1), with one projecting at oral surface, white is less well mineralized in aboral dental plate, including more aboral second set (ov2). Outer dentine (labial and lingual, od) shows developing degrees of mineralization, grey translucent oral, white aboral. Cartilage is also white with low degree of mineralization in both outer layers (ca; jaw tissue in **b**, **c**). Lingual, oral tissue is well mineralized as sclerotic osteodentine (sod), to a depth that reflects the worn surface (white arrow, deep sclerodentine forming front; fields of Fig. 2b; see Fig. 18a for interpretive drawing). **b** adult, worn oral surface with relief from harder dentine of ovoids (ov) and tritors (tri), slender support from mineralized cartilage (ca). **c** juvenile, worn oral surface reveals similar distribution of harder dentine, but as rods (rod; see Fig. 16) and lingually a differently shaped tritor (tri). **d** adult, density dissected μCT render, showing hypermineralized dentine of ovoids and tritors (ov, tri). Scale bars A ,= 0 .5cm, B, D = 0 .5cm. C = 0.25 cm

Our study focuses on dental plates of the extant deep marine species *Harriotta raleighana* (Holocephali; Rhinochimaeridae), with an examination of the dental plates of both adults and juveniles, including anatomy, microstructure and development of the dentine tissue comprising the plates. The dental plates include trabecular dentine supporting lingual tritors (rounded areas of especially wear-resistant material) and oral to aboral (away from the oral surface) series of labial ovoids composed of material comparable to the tritors (earlier called ‘strings of beads’ [5], Fig. 1). There is progressive mineralization of these towards the worn oral, biting surface, at which point all materials are removed by feeding-associated wear. This results in hypermineralized dentine organized as stacks of ovoids, and lingual tritoral pads (shortened to ovoids and tritors hereafter).

We sought to understand how holocephalans develop and maintain functional dentitions without the teeth that characterize not only other chondrichthyans such as sharks, skates, rays (Elasmobranchii), but also stem-group holocephalans such as *Helodus* [7, 8]. Teeth are absent from the embryonic stages of modern holocephalans such as *Harriotta*, and never appear in the development and growth of the complex dental plates. Tooth germs have neither been observed in the oral mucosa around the functional plates of *Harriotta* [6], nor in the early development in *Callorhynchus milii* [5, 9]; instead, there is initially a single blastema for each dental plate, so that separate tooth germs do not contribute to formation of the dental plates.

Essential questions include, how is this constantly growing dentition structured (statodont, all elements retained within the dentition rather than shed and lost) and how are the tissues continuously replaced [1, 4, 10, 11]. We aim to show not only how each dental plate grows, without adding individual replacement teeth, but also how the anatomy of the functional surface is dependent on the developmental growth and arrangement of dentine (Fig. 1a, d; [1, 4]). As teeth are absent, the morphological features of the worn dental surfaces are totally dependent on the relative hardness of each tissue and its arrangement within the plate.

With this background, we have focused on the dental plates of adult *Harriotta raleighana* to investigate developmental renewal within the intrapulpal tissues of the dentition, from aboral to oral, for growth and composition at the oral surface, including all tissues [1, 4]. The *Harriotta* dentition possesses four dental plates on the upper jaws and two on the lower. These plates grow continuously, with feeding-related wear removing dental tissue at the occlusal surface. The hypermineralized tissure (referred to here as HD [5] until we have evaluated its detailed structure) is more wear resistant than other dentine, resulting in areas of harder tissue standing proud on the wear face giving a rough biting surface. Whilst these plates act functionally as teeth, they differ from sets of new teeth developing within the jaw to replace those at the margins. Instead, a super-hard tissue, termed hypermineralized dentine [5] forms deep within a framework of mineralized trabecular dentine, organized as ovoids and tritors. Comparison between adult and juvenile plates reveals the same levels of mineralization in the tritors and ovoids, but with rods in the most rostral parts of the juvenile dentition, with the arrangement of this hypermineralized dentine at the worn surface revealing a different morphology from adult plates.

Our hypothesis is that, despite lacking teeth, there is a regulated addition of the structures made of hypermineralized dentine (ovoids, rods and tritors) that are renewed at the aboral surface, putatively organized by a pattern that is first established in the developing trabecular dentine. Along with this renewal, we also suggest that there is replacement of tritoral tissue aborally and lingually. We will analyze and compare microstructure of the hypermineralized dentine, and investigate how this shows variable mineralization with a mineral composition including magnesium whitlockite, as previously demonstrated for *Chimaera phantasma* [6]. We will trace ratios of calcium and phosphorous to magnesium, across aboral to oral dental profiles, to examine the mineralogy and demonstrate how the mineralization of this supermineralized dentine is substantially different from that known for chondrichthyan teeth. We will consider if the previous terminology is sufficient to characterize all parts of the dental plates, and possibly justify name changes.

## Materials and methods

### Specimen sources

*Harriotta* specimens collected by Marine Research Vessel Scotia (Marine Scotland) in 2015, transferred frozen to Birkbeck and the Natural History Museum, London, where they were defleshed and dried. One adult female was examined, 59.5 cm long (haul 366 RT 15, area 46 d 9, 2000 m depth), along with one juvenile, 32 cm long (no haul data recorded). Additional specimens of an adult and smaller individual were collected by Chris Bird, National Oceanography Centre, Southampton, and previously on a Marine Research Vessel Scotia cruise, some skeletonized.

### Optical microscopy

From serial hard tissue sections through each of the three dental plates present in an adult, photomicrographs were taken with a Zeiss Photomicroscope III in transmitted light (TL); polarized light with polarizer substage to condenser, analyzer slide rotatable (PL), slider for gypsum plate prism at 45° to crossed polars (GP); objectives, × 6.5, × 16, × 40, with prisms for Nomarski differential interference-contrast (DIC); Stereomicroscope Wild MPS45 × 6.5 to × 40 with annular adaptor for Kern Ring Illumination (IL), LED light 60 T-B; both microscopes fitted with a monocular phototube for a CMOS digital camera, Basler (UK) 20,819,762; or a Nikon Coolpix 990.

### Microprobe/EDX

Major-element mineral analyses were conducted using a JEOL JXA8100 Superprobe with an Oxford Instruments AZtec system (EDS) at Birkbeck College, London. Analysis was carried out using an accelerating voltage of 15 Kv, a current of 1 μA, a beam diameter of 1 μm and 40 s acquisition time. The analyses were calibrated against standards of natural silicates, oxides and Specpure metals, with the data corrected using a ZAF programme.

### μCT-scanning

Natural History Museum London: X-ray micro-computed tomography (μCT) was used to examine the skull of adult *Harriotta*, along with upper and lower jaws and dental plates of adult and juvenile individuals (Nikon Metrology HMX ST 225, Image and Analysis Centre). μCT scans were 3D rendered using Avizo (https://www.fei.com/software/amira-avizo/) and Drishti (https://sf.anu.edu.au/Vizlab/drishti/) software.

Kings College London: Specimens for μCT were scanned using a Scanco uCT50 microCT scanner (Scanco, Brüttisellen, Switzerland). The specimens were immobilized in 34 mm scanning tubes using cotton gauze and scanned to produce 6 μm voxel size volumes, using X-ray settings of 70 kVp, 114 μA and a 0.5 mm aluminium filter to attenuate harder X-rays. The scans were automatically scaled at reconstruction using the calibration object provided by the CT manufacturer, consisting of five rods of hydroxyapatite (HA) at concentrations of 0 to 790 mg HA/cm^3^, and the absorption values expressed in Hounsfield Units (HU). The specimens were characterized using the density profile tool of the Parallax Microview software package (Parallax Innovations Inc., Ilderton, Canada), after downsampling to 24 μm voxel size.

## Results

### Adult *Harriotta raleighana* tissue morphology

**Fig. 2:**
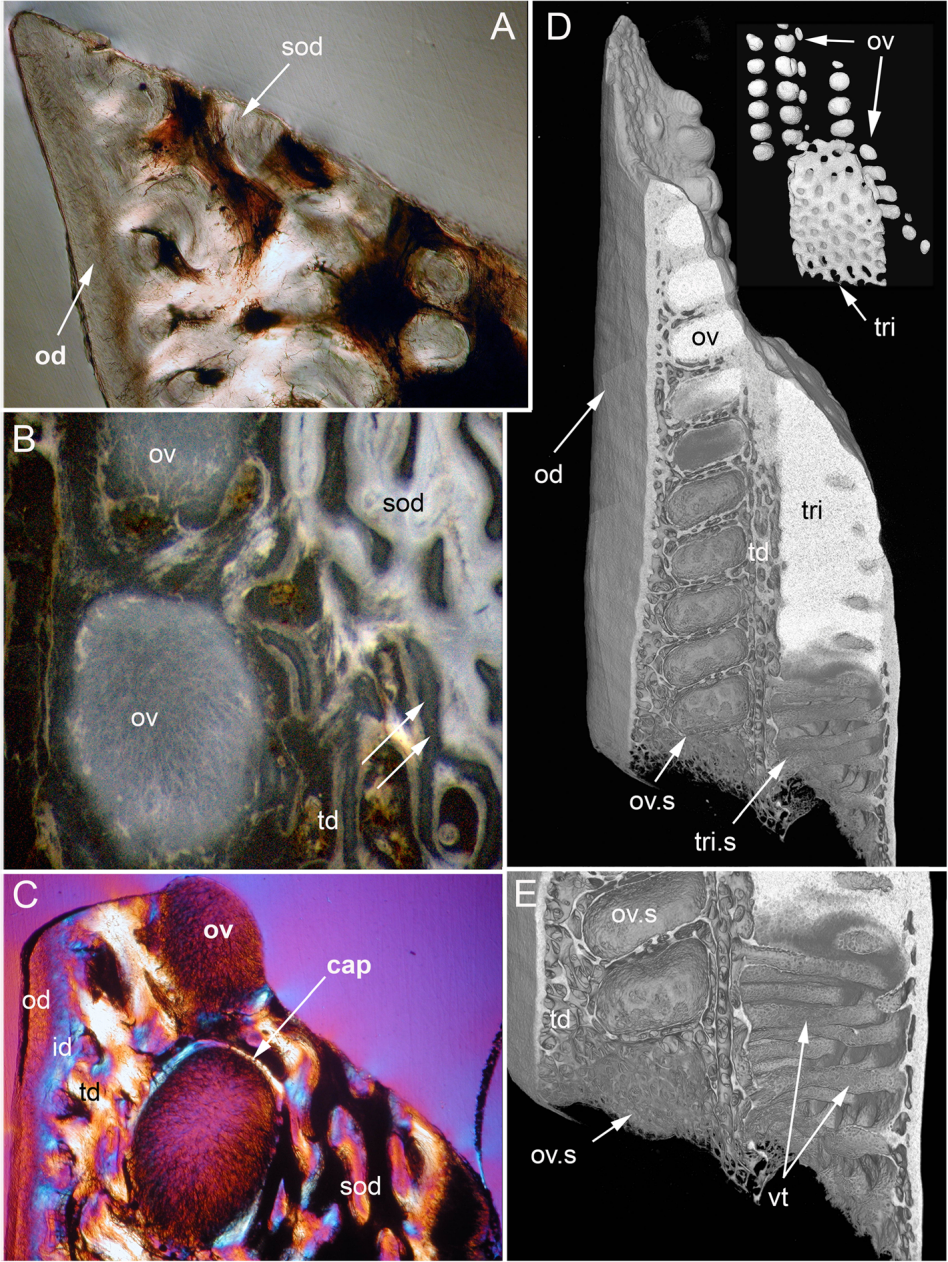
*Harriotta raleighana* (Rhinochimeridae; Holocephali; Chondrichthyes). Microstructure of hard tissue sections in dental plate of a lower jaw alongside virtual μCT. **a–c** Optical microscopy, **d–e** μCT scans: **a** Nomarsky optics of tritoral labial margin without ovoids, worn surface of sclerotic osteodentine (sod); translucent tissue is hypermineralized, for outer dentine layer (od), and most of osteodentine (os) with strong birefringence of organized collagen fibres of trabecular dentine framework (see C), brown colour is partially infilled vascular tissue, and odontoblast tubules. **b** surface illumination of hypermineralized ovoids (grey, ov), region just below oral surface (field in Fig. 1a), sclerotic osteodentine white (see Fig. 1a, sod), trabecular dentine (td) is less mineralized (see D, E for comparable X-ray density), white arrows, deep sclerodentine forming front. **c** field in Fig. 5a, polarized light with a gypsum plate shows opposite orientation of collagen fibre bundles as colours in sclerotic dentine matrix (gypsum plate at -ve 45° to polarizer at 90° gives direction of birefringent tissue components as blue or yellow, not red; see also Fig. 3b, c, Fig. 11e), note the birefringent capsular fibres (cap) enclosing the ovoids, but none inside, only HD tubules. **d**, **e** adult, μCT virtual section through dental plate of tissue arrangement (see Fig. 18b for interpretive drawing), oral surface worn ovoids (ov, top) to aboral (bottom) with sequence of ovoids as mineralized with HD (white) to empty capsules (ov.s) as forming spaces in the trabecular dentine aborally, also tritor spaces organized around the special blood vessels, in parallel tubes (vt, tri.s), **e** close up of aboral margin in Fig. 1d, developing ovoid and tritor spaces between the vascular tubes within the tritor (vt). Scale bar D = 0.25 cm, E = 0 .1cm

The adult dental plates of *Harriotta raleighana* contain two differing arrangements of hypermineralized dentine (HD) in the dental plate, either as separate ovoids arranged in well-ordered series with a ‘string of beads’ appearance (Fig. 1a, b, d, ov1, ov2), or as blocks of tissue in the tritors. The tritors are penetrated by regularly spaced openings for vascular canals embedded in a continuous mass of HD (Fig. 1b, d). The oral surface, produced by feeding-related wear, reflects these two distinct arrangements of the HD, with lingual tritoral pads forming raised areas and separate ovoids forming a series of small bumps arranged labially (Fig. 1b, d, Fig. 2b, c, ov, tri). The tritor in section has almost parallel tubes with vascular connections to vessels in the trabecular dentine, the latter forming the extensive supportive framework in which the ovoids and tritors are situated (td, Fig. 1a, Fig. 2a, b and c). All hard tissues are continually replaced deep to the functional oral surface (aboral, Fig. 1a, Fig. 2d, e), with unmineralized spaces pre-forming in the trabecular dentine prior to ovoid and tritor dentine deposition in them.

The trabecular dentine forms initially as an open framework  (Fig. 2 d, e, ov.s, tri.s) and this becomes more mineralized towards the oral surface as it wears, with spaces being filled later with dentine deposits (sclerotic osteodentine; sod, Fig. 2a, b), but the degree of mineralization of the trabecular dentine remains less than the ovoids and tritors (Fig. 2d). Within the HD of the tritor, the vascular canals are filled with circumvascular dentine of lower mineral density. Levels of mineralization along the series of separate ovoids change rapidly (white least mineralized, grey most, Fig. 1a), demonstrated in hard tissue sections through the dental plate. In these, the older series of ovoids (ov1, nearest to oral surface) are more mineralized than the younger ones (ov2). The most aboral ovoids in each series are the least mineralized (see relative density profiles in ‘Process of mineralization’; Figs. 12 and 13). The trabecular dentine itself becomes more organized and more mineralized, with dense bundles of intrinsic collagen fibres, at the oral surface (see next section; Fig. 1a, Fig. 2a, c). The dental plates form within a sling of polarized collagen fibres of the connective tissue and are fixed to the poorly mineralized cartilage of the jaw (ca, Fig. 1b, c, Fig. 4b and Fig. 15b, c).

### Tissues of the wear surface

In 80 μm serial ground, mounted sections cut vertically through an adult lower dental plate, tissue details of worn (oral) and forming (aboral) regions can be compared within rostral, caudal, labial and lingual regions of the dental plate, with the aboral representing wear-replacing tissue (Fig. 1a, Fig. 7a). Also, density differences of these regions are compared in virtual sections taken from 3D rendered μCT scans (Fig. 2d, e, Fig. 12a, c, and Fig. 13a, c). The trabecular dentine structure forms part of the worn edge of the dental plate, as do the ovoids and tritors, but here at the oral surface the trabecular dentine is consolidated by circumvascular dentine infilling into a dense mineralized tissue, resembling osteodentine in which dense, oriented collagen fibre bundles form (Fig. 1a, Fig. 2b). In the trabecular dentine, the circumvascular dentine becomes more mineralized (sclerotic, Fig. 1a, and Fig. 2a, b and c, sod), to about the same density as the less developed ovoids (Fig. 1a, ov2, see ‘Ovoids as hypermineralized dentine HD’; ‘Tritoral tissue’, ‘Process of mineralization’). The extent of this sclerotic dentine is marked by a deeper junction with the trabecular dentine (Fig. 1a and Fig. 2b, white arrows) and reflects the shape of the wear surface. Also visible are the ovoids, which are proud at the oral surface, and tritors embedded in the trabecular dentine that links all parts to the outer dentine (od) shell of the dental plate, which itself becomes more mineralized (Fig. 1a). All these tissues are renewed at the aboral surface, with the trabecular dentine shaping spaces for newly developing ovoids and continuing the tritors aborally (see Distribution of hypermineralized dentine tissues; Fig. 2d, e, ov.s, tri.s, vt; see ‘Growth as renewal and replacement’).

### Ovoids as hypermineralized dentine HD

**Fig. 3:**
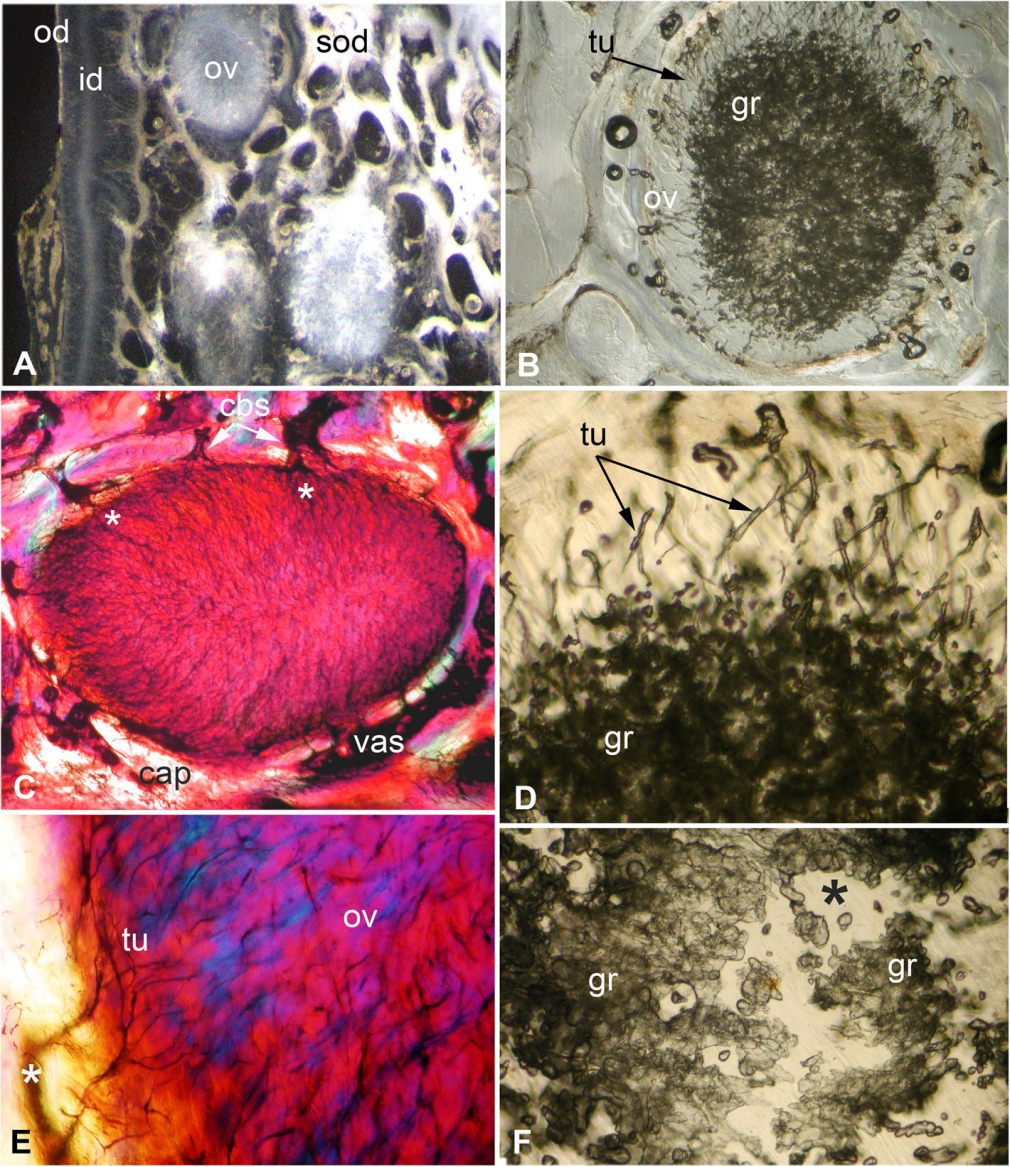
*Harriotta raleighana* (Rhinochimeridae; Holocephali; Chondrichthyes). Microstructure of ovoids using different optical methods to illustrate tissue composition, during their formation. **a** incident reflected light (as in Fig. 1a), ovoids within sclerotic dentine (ov, sod), oral mature hypermineralized ovoid (grey), but two adjacent ones are in transition (white). **b, c** single mature ovoid taken in polarized light with a gypsum plate (see also Fig. 11e), delicate tubules (tu) radiate from the periphery where capsular fibres (cap) surround the whole hypermineralized dentine, also blood vessels (vas) gather here and cell body spaces for odontoblasts (cbs); in C tubules are seen to connect with these peripheral cell body spaces (asterisk), as also in B; the blue colour shows some oriented birefringent structure, early oriented crystals of hydroxyapatite, (see Fig. 9c, d, Fig. 11a, d). **d–f** transmitted light with Nomarsky optics of early ring of formed ovoid tissue (ov) through which tubules run, surrounding a centre of disorganized granular mineral (gr), opaque to light; **e, f** higher magnifications of D, of tubules in peripheral dentine (tu) and F of the centre with saccules (asterisk), tubules and granules in clustered groups (see Fig. 9g, h)

**Fig. 4:**
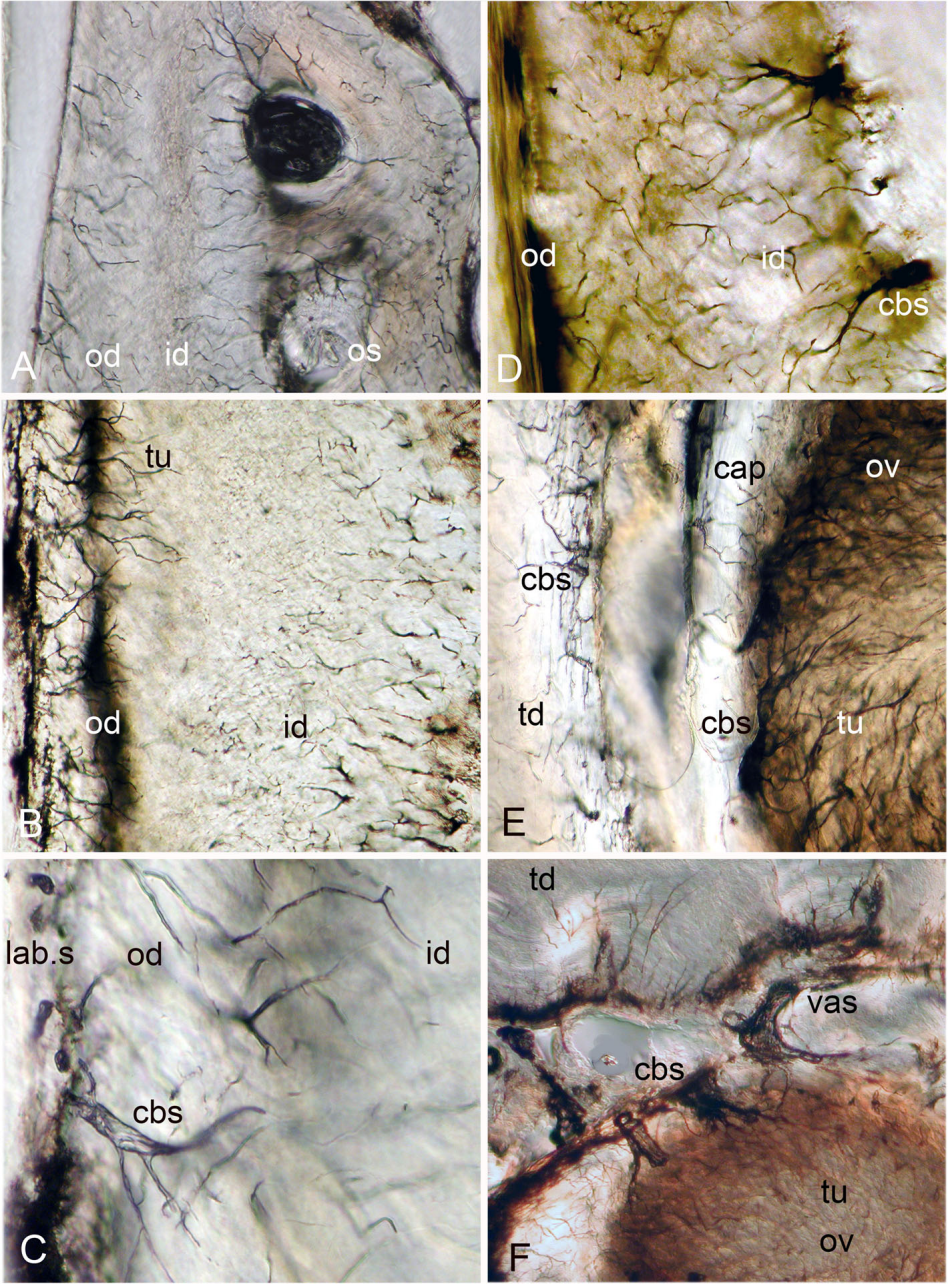
*Harriotta raleighana* (Rhinochimeridae; Holocephali; Chondrichthyes). Microstructure of all dentine tissue, using Nomarsky optical methods to illustrate cellular composition. **a–d** outer dentine (od) and labial surface with cell body spaces (lab.s, cbs), tubules enter from this surface and ramify to link with inner dentine (B, D, id), cell body spaces of inner dentine send tubules (D, cbs, tub) to link with outer dentine. **d** inner dentine matrix has oblique orientation of fibres, from birefringence in Nomarsky (C, D; od, id) shows a sling of collagen fibres to link with the trabecular dentine (td, E, F). **e**, **f** ovoids in mature stages surrounded by vascular canals (vas) crossing the capsular tissue of the trabecular dentine and linked with very large cell body spaces (cbs) providing the source for multitudinous tubules entering the ovoids

The ovoids that project on the oral surface are the most mineralized (translucent grey, Fig. 1a, Fig. 2b, and c, Fig. 12a and d, see ‘BSE ultrastructure’), and not birefringent under polarized light as is the enclosing trabecular dentine (Fig. 2c, Fig. 11d, e and f). All ovoids are encircled by collagen fibres acting as a capsule, as determined by polarized light with a gypsum plate to demonstrate the fibre direction (blue, or yellow, cap; Fig. 2c, Fig. 11e). The ovoids are enmeshed in the trabecular dentine framework, joined to the outer dentine framework of the dental plate (Fig. 2c, Fig. 3a, od) by slings of parallel collagen fibres (Fig. 4c, d). The mineral content of the ovoids is greater than that of the trabecular dentine, as shown by their greater translucency (Fig. 1a, Fig. 2b, Fig. 3a). Multitudinous, very fine calibre tubules ramify radially into the centre of the HD (Fig. 2b, and c, Fig. 3b and c, Fig. 4e and f, tu), linked to wide cell body spaces and with a peripheral ring of blood capillaries (Fig. 3b and c, cbs, vas), as demonstrated using Nomarsky optics (Fig. 4e and f).

The earliest ovoids seem not to have either fibre matrix or tubules early in development (Fig. 3d e and f), and the first to mineralize are white in incident light and black in transmitted light (Fig. 1a and Fig. 3a). Tubules are present in the marginal tissue (Fig. 3b, d and e, tu) and the empty centre is filled with a disorganized mass of granules, in irregular clusters (Fig. 3d and f, gr). Profile graphs that record the relative mineral density changes are described in section ‘Process of mineralization ’, where the sudden increase in mineral deposition of the ovoids is quantified (Figs. 12 and 13).

### Trabecular dentine

**Fig. 5:**
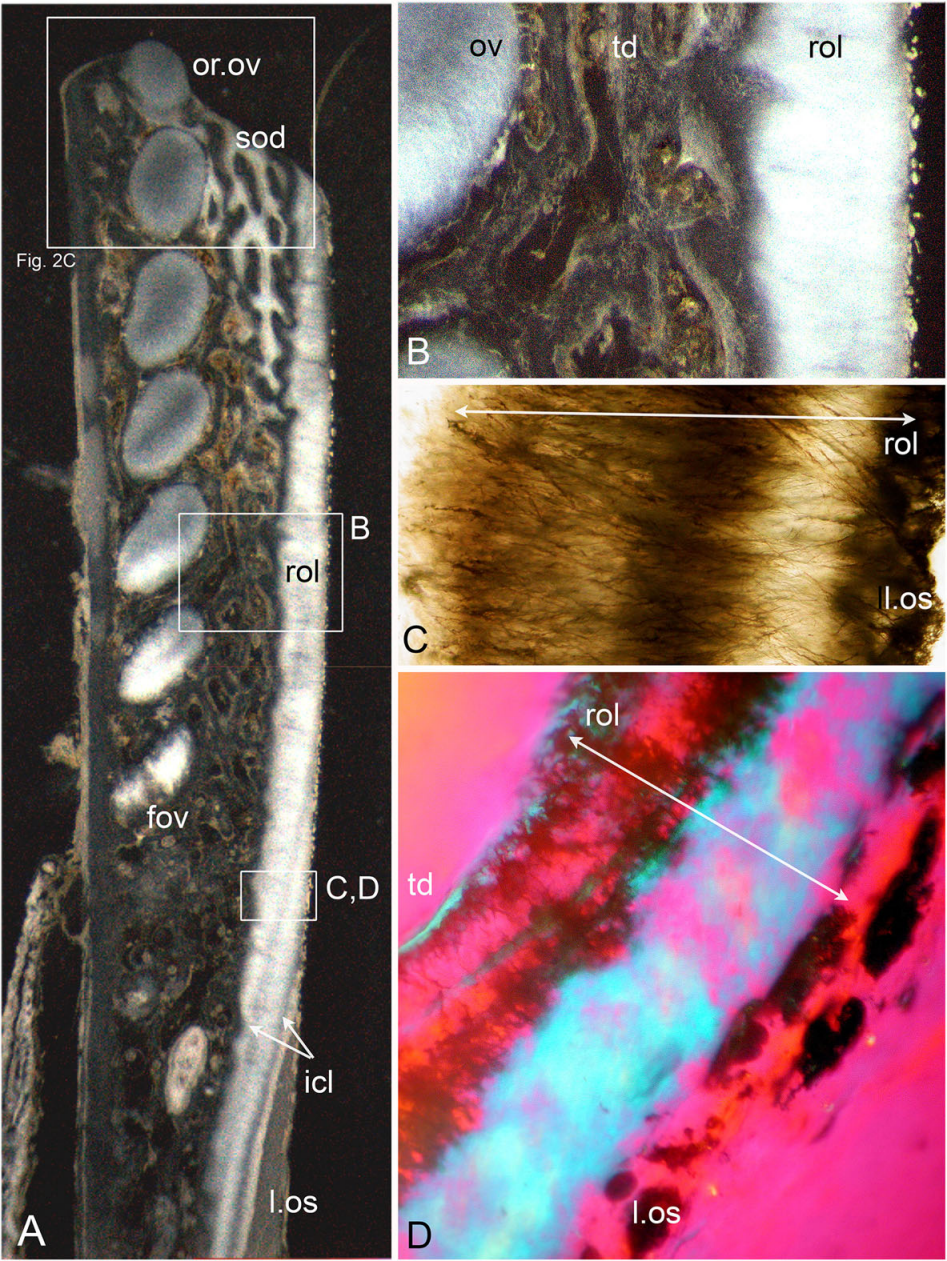
*Harriotta raleighana* (Rhinochimeridae; Holocephali; Chondrichthyes). Microstructure of rostral lingual hypermineralized outer layer using different optical methods to illustrate tissue composition. **a**, **b** micrographs in reflected incident light of hard tissue section from the most rostral end of lower dental plate show position of mineral-dense outer layer on lingual surface (rol, l.os), that is outside all other tissues (field of B in A; field of C, D in A), less hypermineralized than oral ovoids, similar amount to that of sclerotic dentine (sod). Stack of ovoids at oral surface (or.ov) with forming one below, aboral (fov). **c** transmitted light, Nomarsky optics of outer layer, with masses of fine lines crossing each other, may reflect grouping of crystals normal to surface, or fine tubules of mineralizing trioral dentine (see Fig. 6e, f). **d** same in polarized light with gypsum plate (−ve 45°) to show organisation of mineral content, blue suggests normal position of hydroxyapatite crystals, none visible in C, but brown fine lines. Lines in A may be incremental growth from the outer surface (icl)

The entire dental plate is made of dentine, including the supporting outer dentine framework, in the absence of bone, very rare in chondrichthyans ([12] see Discussion). This is evidenced in trabecular dentine along with HD of the ovoids and tritors by polarized cell bodies leading into fine, but networking, tubules within them (Fig. 4). In contrast to osteoblasts, dentine-depositing odontoblasts are characterized by these polarized arrays of tubules within the mineralized matrix, that arise from a cell body located in the soft tissue vascularized spaces, at the edge of the hard tissue (Fig. 4, cbs), but in all trabecular dentine these tubules are arranged as a chaotic network within the dentine matrix. All the trabecular dentine appears to form a framework for ovoids and tritors, linking to the surface layers of dentine via a strongly birefringent band of oblique fibres. The outer dentine layer is a border to each of the symphyseal, labial, lingual and caudal aspects (Fig. 1a, Fig. 2a, b, c, d and e, Fig. 3a, Fig. 4d, e and f). Outer (od, labial) and inner (id, lingual) boundary layers have tubules throughout and connect with those of the trabecular dentine matrix (Fig. 4). Unusually, the labial outer layer (in contact with epithelium, Fig. 1a, Fig. 5a, and Fig. 7a) has wide canals on the outside (presumed to house cell bodies) from which tubules ramify through a collagen fibre matrix inwards to connect with inner dentine tubules of the trabecular dentine (Fig. 4, cbs, tu); this implies a mesenchymal cell-derived tissue on the labial surface of the dental plate that can produce dentine. This outermost dentine also has obliquely oriented, collagen bundles that act as support for the inner trabecular dentine that links to the circumferential fibres around the ovoids, all part of the trabecular dentine (Fig. 2c, Fig.3b, and Fig.4a, e, f). Both outer and inner layers of dentine become more mineralized towards the oral surface and therefore more translucent (Fig. 1a and Fig. 2a).

With increased proximity to the occlusal surface, trabecular dentine becomes dense with collagen fibre bundles and excess mineral to make a resistant structure at the wear surface (termed sclerotic dentine, as a progression from osteodentine) as part of the oral surface (Fig. 1a, sod, Fig. 2a, b and c, Fig. 3a and Fig. 4a; see ‘Tissues of the wear surface’). The change in mineral density of the trabecular dentine, recorded as a profile graph (a relative quantitative measure of μCT density, Hounsfield Unit (HU) value) is half that of the HD ovoids and tritors (Fig. 12b and d). The distribution shows that progressive mineralization of the trabecular dentine profiles the degree of wear at the oral surface as the advancing front follows the shape of the worn surface (Fig. 1a and Fig. 5a, white arrows, Fig. 12a and b).

### Hypermineralized rostral lingual layer

**Fig. 6:**
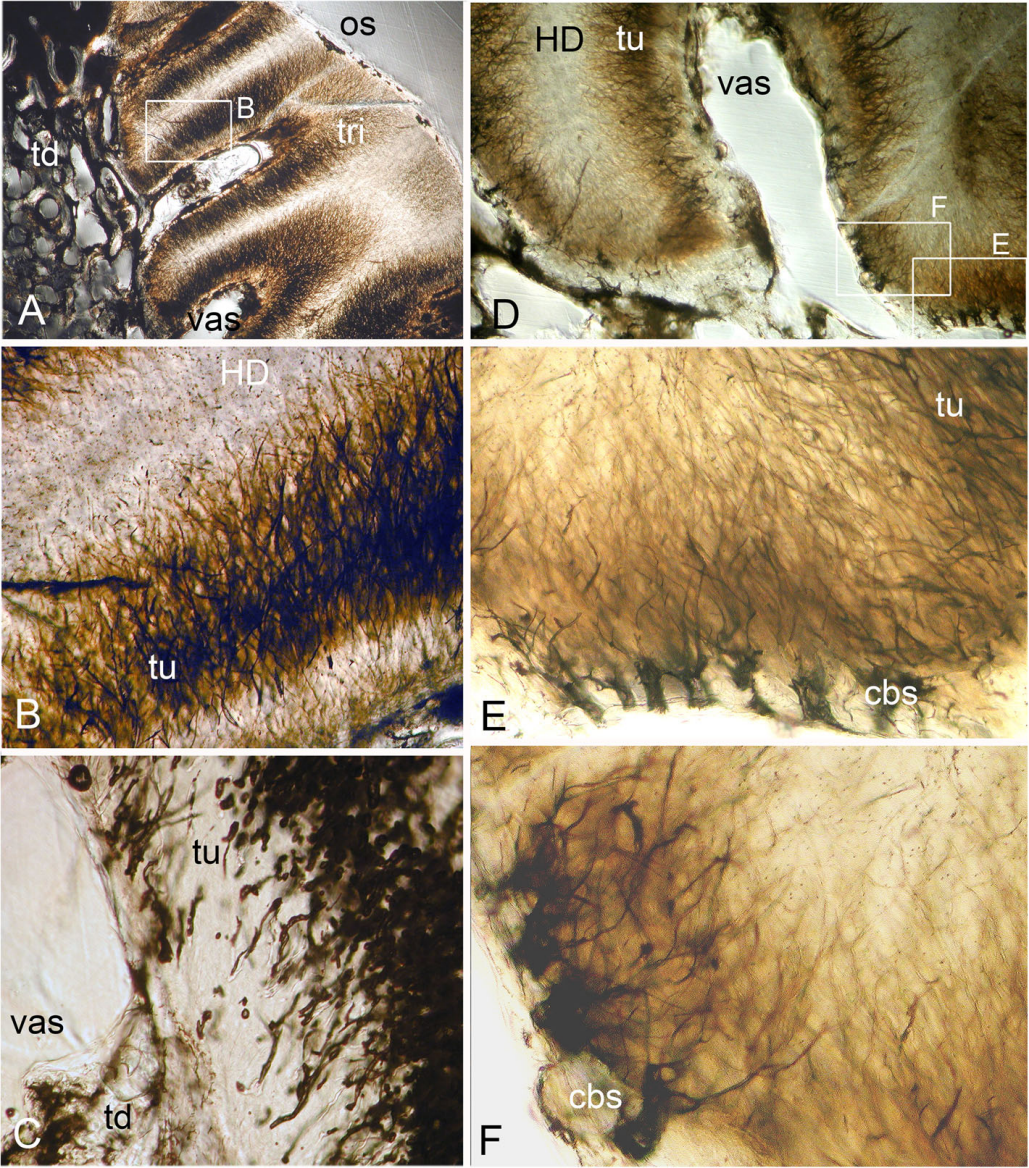
*Harriotta raleighana* (Rhinochimeridae; Holocephali; Chondrichthyes). Microstructure of lingual tritor with optical microscopy to illustrate tissue composition. **a**–**f** Nomarsky optics of tritoral tissue (tri), **a**, **b** at the oral surface (os), **c–f** deeper in at the vascular surface (vas) to compare with ovoids (Fig. 3). Dark areas are a mass of tubules (tu) branching (E, F) from very large cell body spaces (cbs) running far into the hypermineralized region, in **b**, **e**, **f** finest tubules are seen resolved at these magnifications. **c** earlier stage of tritor mineralization at the forming surface where tubules run into an opaque mass of mineral and vesicles, or saccules, and wide tubules, full of the pre-mineralization phase material (see Fig. 10a, b, c, d, e, f, g, h and i)

**Fig. 7:**
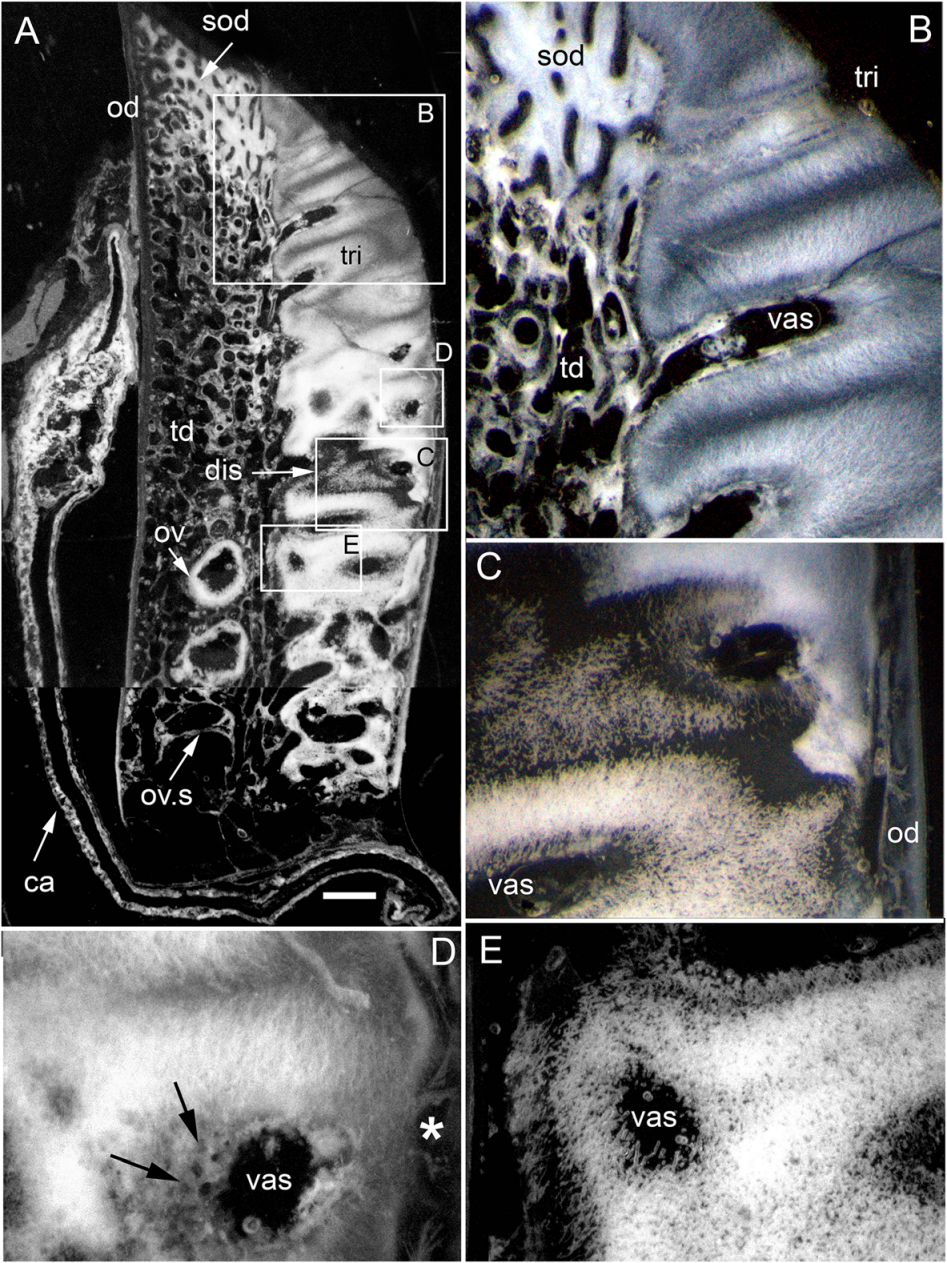
*Harriotta raleighana* (Rhinochimeridae; Holocephali; Chondrichthyes). Discontinuity of tritor in aboral region, caudally, optical microscopy in reflected surface illumination. **a–e** at increasing magnifications × 6.5 –× 16. **a** whole section of dental plate in mineralized labial cartilage (ca), outer dentine in contact at the oral margin, shows separation of upper, highly mineralized (grey) tritoral region (tri), with smooth worn surface joining with sclerotic dentine (sod), from lower mineralizing aboral tritoral tissue. Lower mineralized, aboral region, is separated by a mineral-absent line (dis, arrow) from the oral tritor, this disjunct region in field of C. **b** at higher magnification of tritor and sclerotic osteodentine from field in A. **c** high magnification (field in A), disjunct region separates old, more oral tritor from new, more aboral, all beneath a translucent layer of outer dentine (od). **d** Field in A, of upper tritoral tissue, above the disjunction, linear mineralization of hypermineralized dentine of intervascular matrix, with less dense mineralized dentine around vascular canal (perivascular), holes for entry of tubules (black arrows) and nutrient supply from vascular outer dentine (asterisk). In contrast **E** (field in A), is part of the new tritor region below the disjunction, with granular mineralization and vascular canal without circumvascular dentine, reflecting the incident light (intense white). Scale bar A = 0.5 mm

Exceptionally, in the most rostral part of the dental plate (symphyseally), there is a thick, highly mineralized layer with many properties of the HD of tritors and ovoids, but without enclosed vascular tissue as in the former (Fig. 5, rol). In incident light this thick layer is white, as are the forming ovoids (Fig. 5a and b, fov) and so has not become as translucent as the most highly mineralized ovoids near the oral surface (Fig. 5a, or.ov). Throughout, dark brown lines within the tissue, similar to tubules in the tritor (Fig. 6d and e) but different in that all are normal to the surface and thicker, run at angles to each other in the inner layers (Fig. 5c). This rostro-lingual layer directly abuts the trabecular dentine (Fig. 5b, trabecular dentine). In polarized light it has a diffuse birefringence that is positive with a gypsum plate (blue in the NE quadrant, Fig. 5d), interpreted as crystals at right angles to the surface, not seen in the other HD. There is at least one incremental layer line at an acute angle to the surface (Fig. 5a, icl), lines on the surface of an intact dental plate have also been observed. None of the more caudal regions of the sectioned dental plate show this layer (Fig. 1a and Fig. 7a).

### Tritoral tissue

**Fig. 8:**
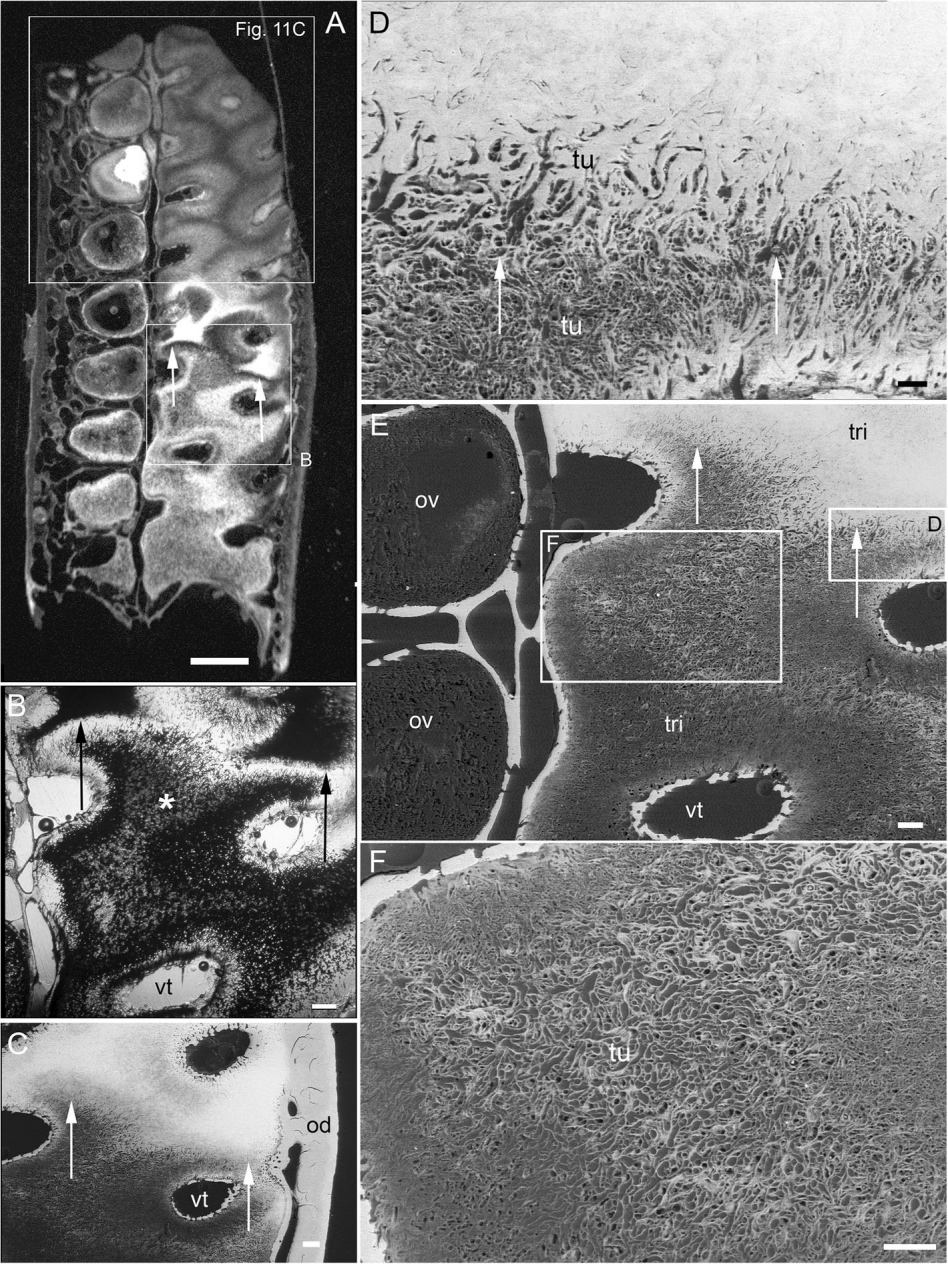
*Harriotta raleighana* (Rhinochimeridae; Holocephali; Chondrichthyes). Discontinuity in tritor of caudal part, upper plate reflected and transmitted light with BSE. **a**, **b**, optical micrographs, c**–f**, BSE of polished section as scanning electron micrographs. **A** incident reflected light (× 6.5), labial ovoids alongside lingual tritor with distinct disjunct region below the functional tritoral region (white arrows), including fields shown in B and Fig. 11c. **b** tissue details shown with transmitted light, masses of tubules (asterisk) and dense light-obstructive areas, below line indicating disjunct region (black arrows). **c** below disjunct line (white arrows) very low mineralization, separated from the high mineral density above. This line does not cross into outer dentine (od) at lingual surface. **d, e** disjunct line (white arrows) with tubule masses below, high mineral density above, higher magnifications of fields (D, F) shown in E. **d** tubules (tu) below the disjunction, and fewer above in the high mineral density region. **e** pre-mineralized ovoid spaces (ov) adjacent to mineralized tritor (tri) with dentine lining vascular tubes (vt). **f** chaotic tubules, multiple sizes, with vesicles and saccules (compare with Fig. 9). Scale bars A = 0 .5mm; B, C, E, F = 100um; D = 30 μm

**Fig. 9:**
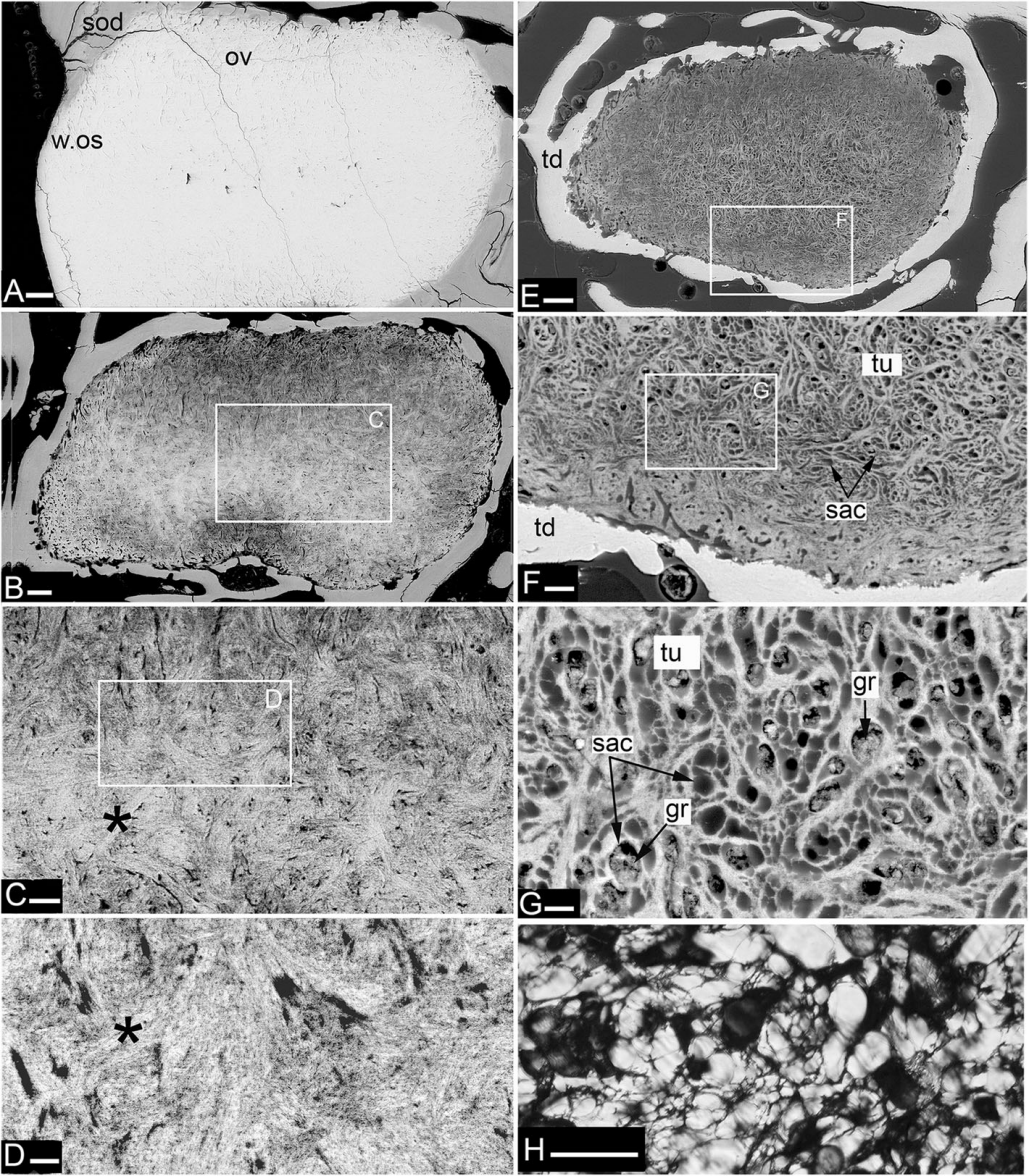
*Harriotta raleighana* (Rhinochimeridae; Holocephali; Chondrichthyes). Progressive mineralization of ovoid tissue with BSE ultrastructure lowest to highest magnification. A hypermineralized, featureless dentine filling ovoid at worn oral surface (ov, w.os), surface loss of sclerotic dentine (sod), compared to **e**, representing one of the least mineralized ovoid. **b** mineralization beginning in centre of ovoid, B and E are more aboral compared to A, field of C shown. **c**, **d** showing the more linear arrangement of the mineral alongside the tubule walls in the centre of the ovoid (asterisk), with tiny empty tubule lumen, the mineralized periphery of the ovoid is slightly less dense dentine, field of D shown. **d** mineralization shown as crossing crystal bundles, reflecting original chaotic mix of the dentine tubules, individual crystals are not resolved. **e** barely mineralized ovoid, within narrow mineralized trabecular dentine capsule (td), central region of open tubules as shown in F. **f** closeup of tubules (tu) with open saccules (sac), field of G shown. **g** matrix of ovoid filled with complex network of dentine tubules (tu), with enlarged, widened tubules, saccules, (sac), tiny open tubules, vesicles, and empty spaces, some have small mineral granules (gr) within. **h** slightly lower magnification than G of forming ovoid region, but in transmitted light with Nomarsky optics, to show the same chaotic collection of fine membrane vesicles, thicker saccules, some optically dense material in them (disorganized mineral) and background faint linear structure. Scale bars A, B, E = 100 μm; C, F = 30 μm; D, G = 10 μm; H = 50 μm

**Fig. 10:**
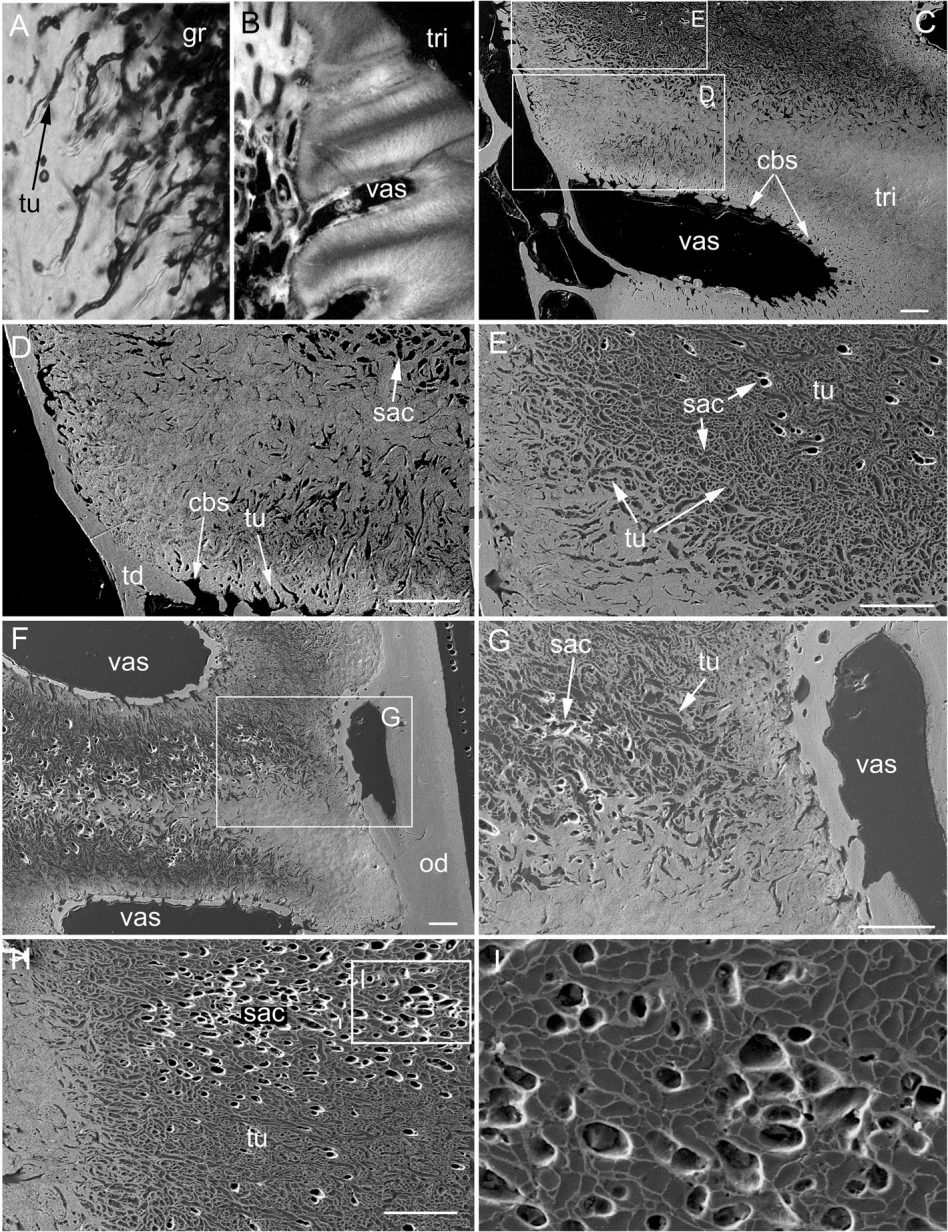
*Harriotta raleighana* (Rhinochimeridae; Holocephali; Chondrichthyes). Progressive mineralization of tritor tissue, microstructure and ultrastructure lower plate, low to high magnification. **a** Nomarsky optics at forming front of tritoral dentine (see Fig. 6a), where multiple wide, and narrow tubules run into dense mass of saccules, and granular crystals (tu, gr). **b** most highly mineralized (translucent) dentine at worn tritural surface (tri, see Fig. 7a, b). **c–h** (electron micrographs at progressive, consistent magnifications × 250, × 500, × 1000). **C** mineralized tritor (tri), with many cell body spaces (cbs), tubules running from these into HD, differential mineralization (lighter indicates higher mineralization), same level as circumvascular dentine and trabecular dentine, fields of D, E shown. **d** saccules (sac) in low mineral density region. **e** chaotically arranged tubules (tu) in less mineralized part of tritor, saccules within these (sac). **f, g** more aboral, stage earlier in process of mineralization than in C, D. **f** mineralization is highest in the middle of the tritor, between two vascular canals, field of G is shown, beneath outer dentine (od, in F) including vascular canals (vas) that supply the hypermineralized dentine. **g** extensive volumes of disorganized dentine tubules (tu), with saccules (sac), many saccules are empty with white edges, shown in relief (edge artefact of BSE). **h** region of tritor dominated by complex network of tubules in large numbers, many as expanded saccules, field of I shown. **i** transmitted light, Nomarsky with closeup of saccules (see also Fig. 9f, g, h). Scale bar A, C–H = 100 μm; B = 300 μm; I field width is 150 μm

**Fig. 11:**
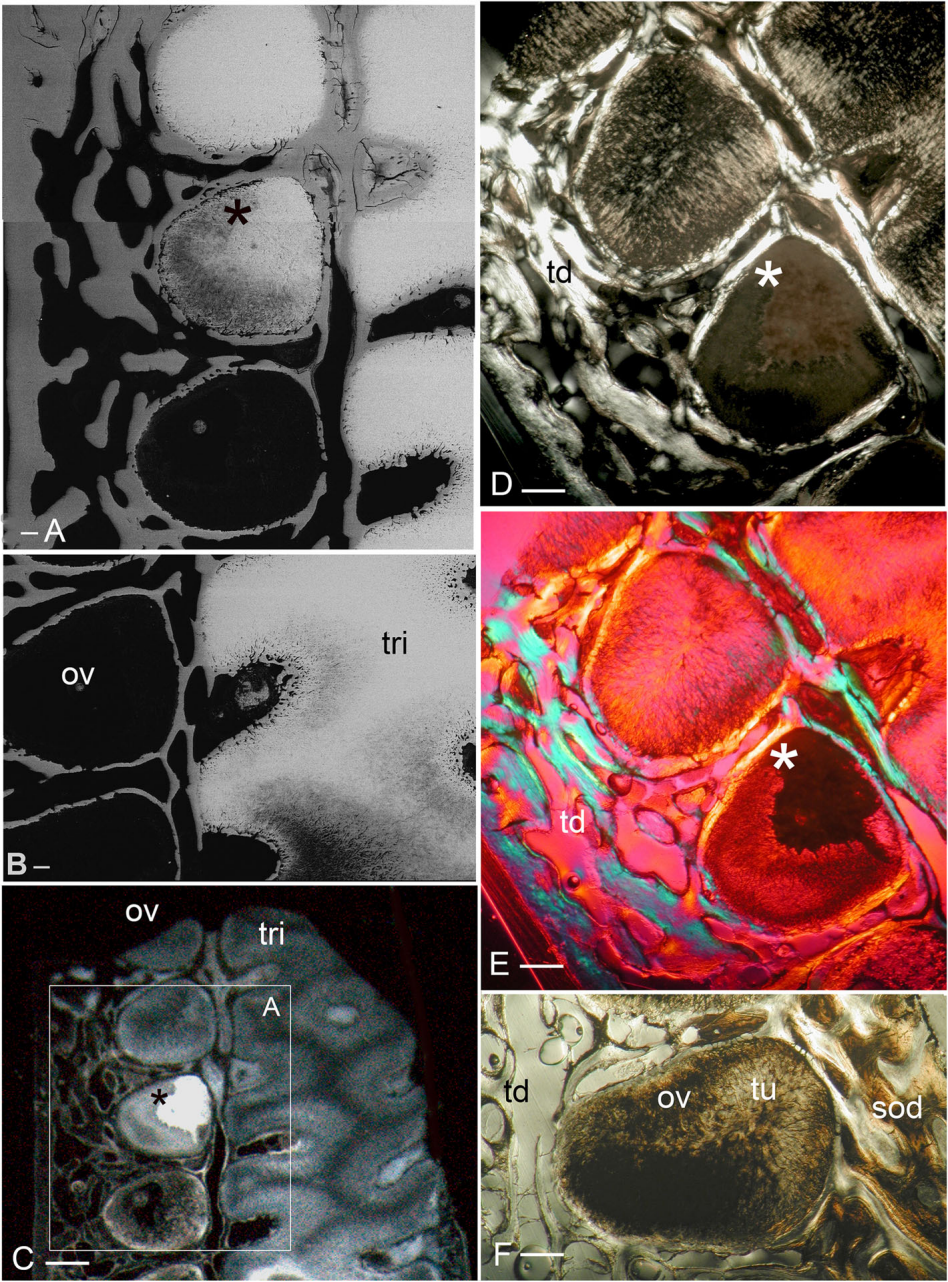
*Harriotta raleighana* (Rhinochimeridae; Holocephali; Chondrichthyes). Comparison of same partially mineralized ovoids, in μCT and optical microscopy, upper caudal dental plate. A BSE from field in C, photomicrograph in incident reflected light of the ovoids (ov) and tritor (tr) at oral surface, the same partially mineralized ovoid (*) is presented in four different modes of visualization (**a, c–e**), field in Fig. 8a. **b** BSE, aboral to **A**, ovoids (ov) lacking mineralization and dentine tubules, tritor with differential mineralization of hypermineralized dentine more orally, but less mineralization aborally. **d, e** photomicrographs in polarized light, E with gypsum plate added, ovoids at angle of 45° to polarisers, shows strong birefringence of trabecular dentine (td) (NE blue, NW yellow in quadrants between polarizer and analyzer); note in **D** most mineralized ovoids and tritor are weakly birefringent, but as linear birefringence at right angles to each other and with very faint blue and yellow (gypsum plate inserted at 45° to P and A data not shown), whereas ovoid with * is not birefringent, indicating a major change in form of mineralization. **f** Nomarsky optics, ovoid with an opaque area (black) similar to *, within the sclerotic dentine (sod, close to worn surface at far right) that is at the same mineral phase as ovoids marked by *, but tubules (tu) are visualized in the mineralized part by this method. Scale bar A, B, C, =100 μm; D, E, F = 50 μm

**Fig. 12:**
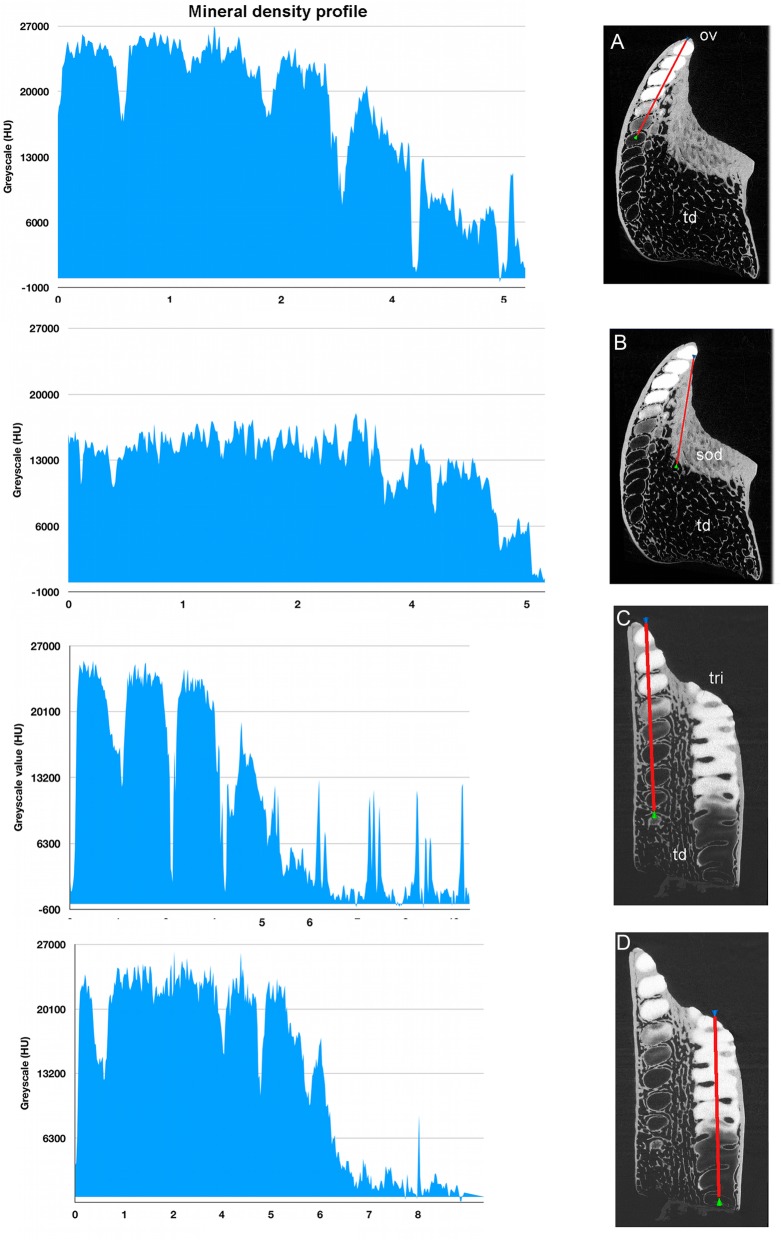
*Harriotta raleighana* (Rhinochimeridae; Holocephali; Chondrichthyes). Calcium (Ca) density profiles relative to HAP standard, on virtual sections in μCT, adult lower plate. Density profiles measured in Humboldt units (HU), blue graphs, left. **a**–**d** virtual sections through dental plate, density measured along red line from oral to aboral. **a** rostral labial set of ovoids (ov), hypermineralized dentine (HD) from oral high density to aboral no density. **b** profile through adjacent sclerotic dentine (sod) is half numerical density value of HD, lowest in trabecular dentine (td) aborally. **c** labial caudal ovoids including many unmineralized ovoid shapes, with very low values for relative density, adjacent to mineralized tritor (tri). **d** profile of tritoral HD with developing aboral tritor with low HU values and sudden change to higher ones. All regions with hypermineralized dentine have the same values for relative Ca density

The punctate surface of the lingual pads (Fig. 1b and d) is formed by blood vessels deep within the tritor, forming more and more circumvascular dentine of slightly lower mineral density, this density equivalent to that of sclerotic trabecular dentine (see ‘Tissues of the wear surface’, Fig. 2d, Fig. 6a and d, Fig. 7a, c and d). This activity fills in the vascular canals completely (vas, Fig. 7d and e, as each develops a lining of pulpal cellular tissue) when reaching the surface, but has a lower mineral density so wears to leave characteristic depressions (punctate surface). The highest mineral density of the tritor is at the oral surface (e.g., Fig. 2d), grey in incident, white in transmitted light (Fig. 6a, Fig.7a and c, and Fig. 11b; see ‘Process of mineralization’, relative measure of Ca density, Fig. 12). The vascular pulp canal within a region of active mineralization of the HD is lined first by joined cell body spaces (Fig. 6c, d e, and f), then by normal dentine linked to the trabecular dentine (Fig. 6a and d) and encloses the wide cell body spaces that give rise to multitudinous, fine branching tubules (cbs, Fig. 6e and f, vas, Fig. 7d and Fig. 10). These branches of the odontoblasts penetrate the tritor tissue completely, into the region of highest mineral density (Fig. 6c, HD; b, d, e, f, white; a, grey; Fig. 7a and b), as also observed within the ovoids (see ‘Ovoids as hypermineralized dentine HD’), where cells are adjacent to blood capillaries. In forming regions, the lining dentine is not present around the vascular canals, best seen in surface illumination, where the crystals are still forming in the tritoral HD (Fig. 7a, d and e, arrows). The ultrastructure shows the vast numbers of tubules in the pre-mineralizing tritoral regions (Fig. 8d, e and f, Fig. 10d, e, f, g h and i), also in the ovoids (Fig. 9f, g and h: all BSE images are in §1.8, Figs. 8 and 10).

### Growth as renewal and replacement

#### Renewal

**Fig. 13:**
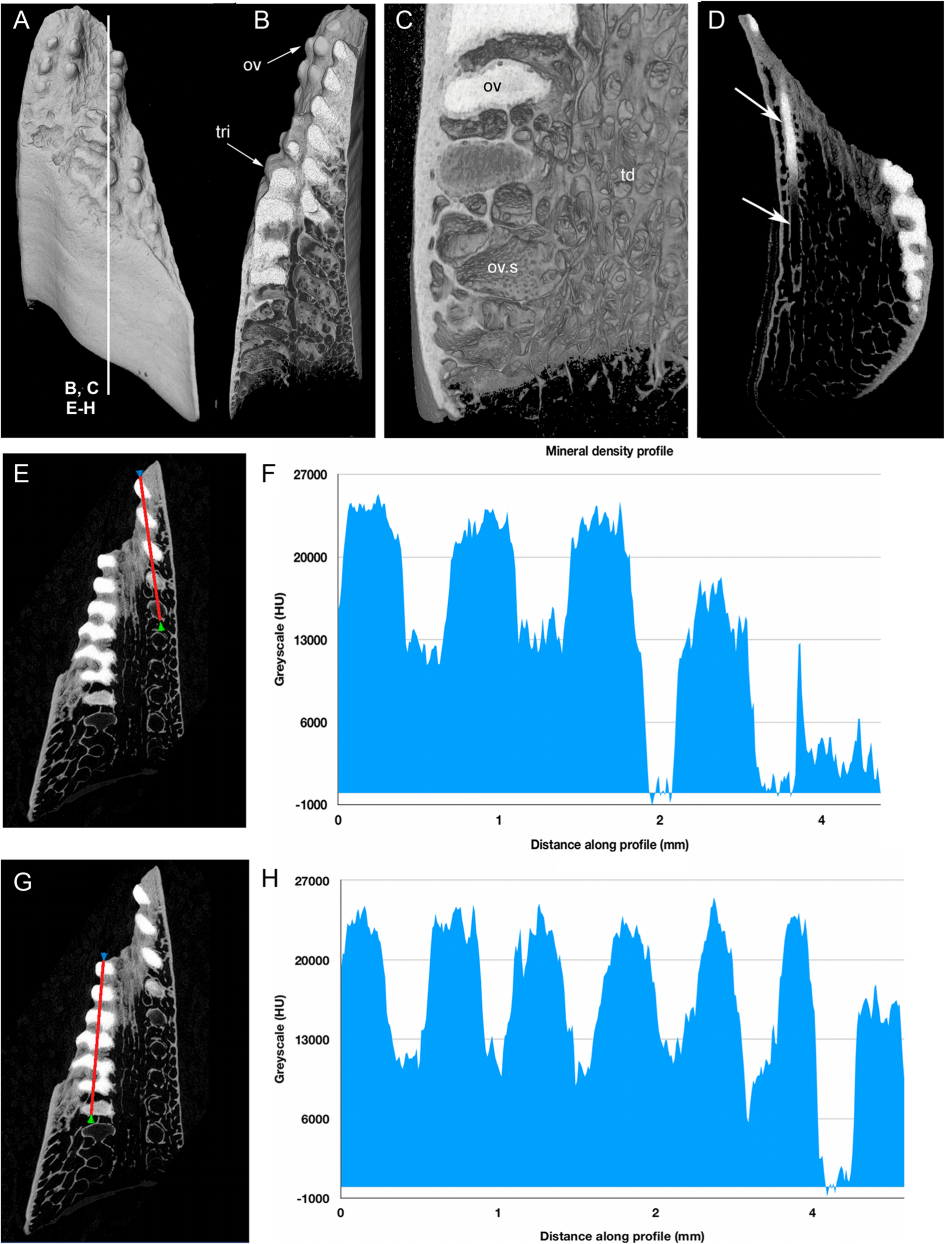
*Harriotta raleighana* (Rhinochimeridae; Holocephali; Chondrichthyes). Calcium (Ca) profiles relative to hydroxyapatite standard, on virtual sections in μCT, juvenile lower plate microanatomy. Density profiles measured in Humboldt units (HU), blue graphs (**f**, **h**). Labial is to the right in all these μCT images. **a** dental plate rendered showing worn oral surface with hypermineralized dentine (HD) of ovoids and a lingual tritor projecting, these of a different shape to the adult (Figs. 1, 16, 17). **b**, **c**, virtual sections through A, labial ovoids (ov) and lingual tritor (tri) with C close up of B, show preformed shape of all HD in the trabecular dentine (td) of the aboral regions (ov.s). **d** virtual horizontal section through tritor and unmineralized rod (white arrows), showing same aboral-oral mineralization as in ovoids and tritors. **e**, **g** virtual sections through dental plate, density measured along red line from oral to aboral. **f**, **h** profiles of ovoids in **E**, tritor in **F** with same HU values for the most dense HD at the oral surface and lower values for the trabecular dentine between these, with little difference to those recorded for the adult plates (Fig. 12a, b, c and d)

The process of trabecular dentine formation starts at the aboral surface within the soft tissues forming the dental plate, that sits within a depression in the jaw cartilage (Fig. 1c; Fig. 16a, b and c, g, asterisk). Growth of outer and inner trabecular dentine starts to form the framework for the dental pulp tissues from which HD later forms as ovoids and tritors, secreted by specialized odontoblasts (see Discussion: ‘Cells involved in secretion of dentine’, where a new name is proposed for cells producing the hypermineralized dentine HD, previously called pleromoblasts; Figs. 3 and 6; see ‘Ovoids as hypermineralized dentine HD’, and ‘Tritoral tissue’). The spaces for ovoids and tritors (apparently empty as lacking density in μCT scans, but may contain soft tissues) appear within the trabecular dentine before any obvious mineralization, or any matrix formation (Fig. 2d and e). With BSE imaging on a sectioned surface the change within the ovoid stack from empty to partial mineralization occurs in the aboral to oral direction (Fig. 8e, ov, Fig. 11a, b and c). Also with surface illumination of hard tissue sections, the more aboral HD of the ovoids in the stack is white and not grey as is the less hypermineralized aboral tissue of the tritoral lingual pad (Fig. 1a, ov2, Fig. 7a, b and d). This rapid change to higher levels of mineralization was evaluated by Ca profiles of each region in aboral to oral scans on virtual density rendered sections in μCT, for relative density values (‘Process of mineralization’, Figs. 12 and 13). The distribution of HD emphasises this rapid change from mineral free to fully mineralized ovoids and tritoral pulp canals, stacked within the trabecular dentine framework (Fig. 16b, c d and e). A schematic diagram depicts the distribution of all tissue types from forming aboral to functional oral, in sections from rostral to caudal positions along the lower dental plate (see ‘Summary of adult dental plate structure**’**, Fig. 18).

#### Replacement

in the more caudal sections of the dental plates, incident light reflection reveals an abrupt disjunct region within the lingual tritor pads, representing a change between less mineralized, and less organized tissue, to translucent HD of the more oral, functional tritor (Fig. 7a, c, dis). As discussed further below, including the ultrastructure, this could be a second, new tritoral region, abutting the older one that is part of the oral surface, and thus representing its replacement. A similar disjunct region in the tritoral tissue is present within the posterior plate of the upper jaw, in the same specimen, indicating a separate, less mineralized tritor distinct from the more mineralized functional tritor (Fig. 8a,b, c, d and e, arrows). The sudden change at the disjunction was obvious with the features of lowest mineralization (Fig. 8b, asterisk) equal to that in the last–to-form region of the tritor above the arrowed line. This replacement region also has a new series of ovoids alongside, labial to the disjunct tritor, in early mineralization phase (Fig. 7a, ov; see ‘Process of mineralization’). In BSE images of the same serial sections, both these regions show this disjunction (Fig. 8b, c d and e, arrows), separating highly mineralized HD from the much less mineralized new tissue. All of the proposed new tritor is forming inside the lingual outer dentine, so the separation would not be observed on the outer surface without section views.

The immature ovoids with partial mineralization around the periphery (white rings) and empty centres are located above empty oval spaces in the forming aboral trabecular dentine (Fig. 7a, ov.s). However, ovoids are not present directly below the functional oral surface (instead sclerotic osteodentine is present; Fig. 7a, sod) suggesting the immature ovoids are a new series of ovoids, comparable to the new tritor series, also forming in the caudal part of the dental plate. Whereas in the rostral section of the plate, where only ovoids are present (Fig. 1a, d), it is also striking that a separate aboral series of early mineralizing ovoids (white, ov 2) are forming well below the translucent, well mineralized ovoids (grey, ov 1), that function at the oral surface as the hardest tissue present (Fig. 1a, box).

### Visualization of ultrastructure by backscattered electron miscroscopy

To investigate the ultrastructure of the mineralization during formation of HD we used BSE on the sectioned polished surfaces from the histological study so that direct comparison was possible (Figs. 3, 6 and 9h, Fig. 10a, b). We compared development in ovoids and tritors, from those at the oral surface with the greatest mineralization, to those with the least in aboral regions (Fig. 9a, b, c, d, e, f, g and h, Fig. 10d, e, f, g, h and i). In both ovoids and tritors, we observed the same features at each stage of the mineralization process; and at the disjunct separation between an old tritor and a new one, the sudden change at this disjunction was clear (Fig. 8d, e, Fig. 8b, f). Ovoids that were apparently empty of mineral and negligible matrix compounds are encapsulated within their own peripheral dentine, that has the same mineral density as equivalent to the dentine bordering the tritoral surface and in the lingual outer dentine (Fig. 8e, ov, od). Similarly the vascular canals of the tritors were lined with this ordinary dentine, all more mineralized than early stages of low mineral HD (Fig. 8c, d and e, Fig. 10b, c, d, e and f).

There are multiple, enlarged, tubules entering into the tritoral matrix from the vascular and trabecular dentine margins (Figs. 6 and 10a, b), also into the ovoids from the capsular dentine (Fig. 3b, Fig. 4c). These tubules form an intricate, complex, maze of widened tubules in the low mineral sites (Fig. 8b, d, e and f, Fig. 9e, f and g, Fig. 10c, d, e, f, g, h and i), with some small vesicles, and expanded membranous saccules, as seen also in Nomarsky optics as a direct comparison (Fig. 10h). Some optically opaque saccules could contain an amorphous mineral phase (see ‘Process of mineralization’), others are open with granular mineral inside (Fig. 3b, d, f, Fig. 8f, Fig. 9e, f and g, Fig. 10e, f, g, h and i). Those ovoids with intermediate mineralization (Fig. 9b, c and d) had a dense mineral centre (comparable to the encapsulating dentine) where arrangements of mineral were linear (Fig. 9c, d), some alongside tubules, with far fewer saccules, except in low mineral border regions (Fig. 9b, c and d).

Quantitative levels of calcium mineralization are discussed in the next section (‘Process of mineralization**’**, Figs. 12 and 13) from Ca profiles given as grey scales in units relative to hydroxyapatite across a defined line that relates to these described structures.

### Process of mineralization

All methods (Nomarsky optics, PL, SI, Ca/ P concentration, Drishti segmentation, EDX elemental maps) demonstrate that HD tissue becomes mineralized only closer to the oral surface of the plate (Fig. 1a, Fig. 2d, e) and at a level that exceeds that of the surrounding trabecular dentine.. This pattern also occurs in juvenile dental plates, where the levels of Ca-based mineral in the HD are equivalent to that of adults (Figs. 12 and 13). BSE electron images (Fig. 11a) are compared directly with those from optical methods for identical ovoids in the same section (Fig. 11b, c, d and e, asterisk). In these, one ovoid is at a transitional stage and has a portion with very low electron density (Fig. 11a, asterisk), shows no birefringence (Fig. 11d, asterisk), is opaque to transmitted light (Fig. 11e, asterisk), and scatters light with surface illumination (Fig. 11c, white region, asterisk). All these characters suggest a labile non-crystalline mineral rich phase. Another ovoid of a similar stage, close to the worn surface below the sclerotic dentine, also seems to have a refractile deposit of non-crystalline material (Fig. 11f, black region) and is not birefringent, also odontoblast tubules occur within.

In all Ca density plots (Fig. 12a, c, d, Fig. 13f, h), there is a sudden elevation from the apparently empty ovoids and tritor spaces, to a mineral density about half the level of fully mineralized, to maximum mineralization (from 6300, through 13,000 to 27,000 HU (Hounsfield units); Fig. 12a; rostral ovoids; c, caudal ovoids, d, tritor). By comparison, a profile through sclerotic osteodentine shows the relatively lower density of this tissue that forms below the functional surface (13,000 HU, Fig. 12b; histology, Fig. 1a, Fig. 7d, Fig. 17). In these profiles, the lowest density of the more aboral ovoids and tritors is evident, even the trabecular dentine around the spaces has a higher value than these, but later in the mineralization process this is reversed (4th ovoid from oral surface, Fig. 12c), also true for the juvenile plate (Fig. 13e, f). In both juvenile and adult plates the distinction between partially developed ovoid and tritor is more distinct, and the trabecular dentine appears much less mineralized.

**Fig. 14:**
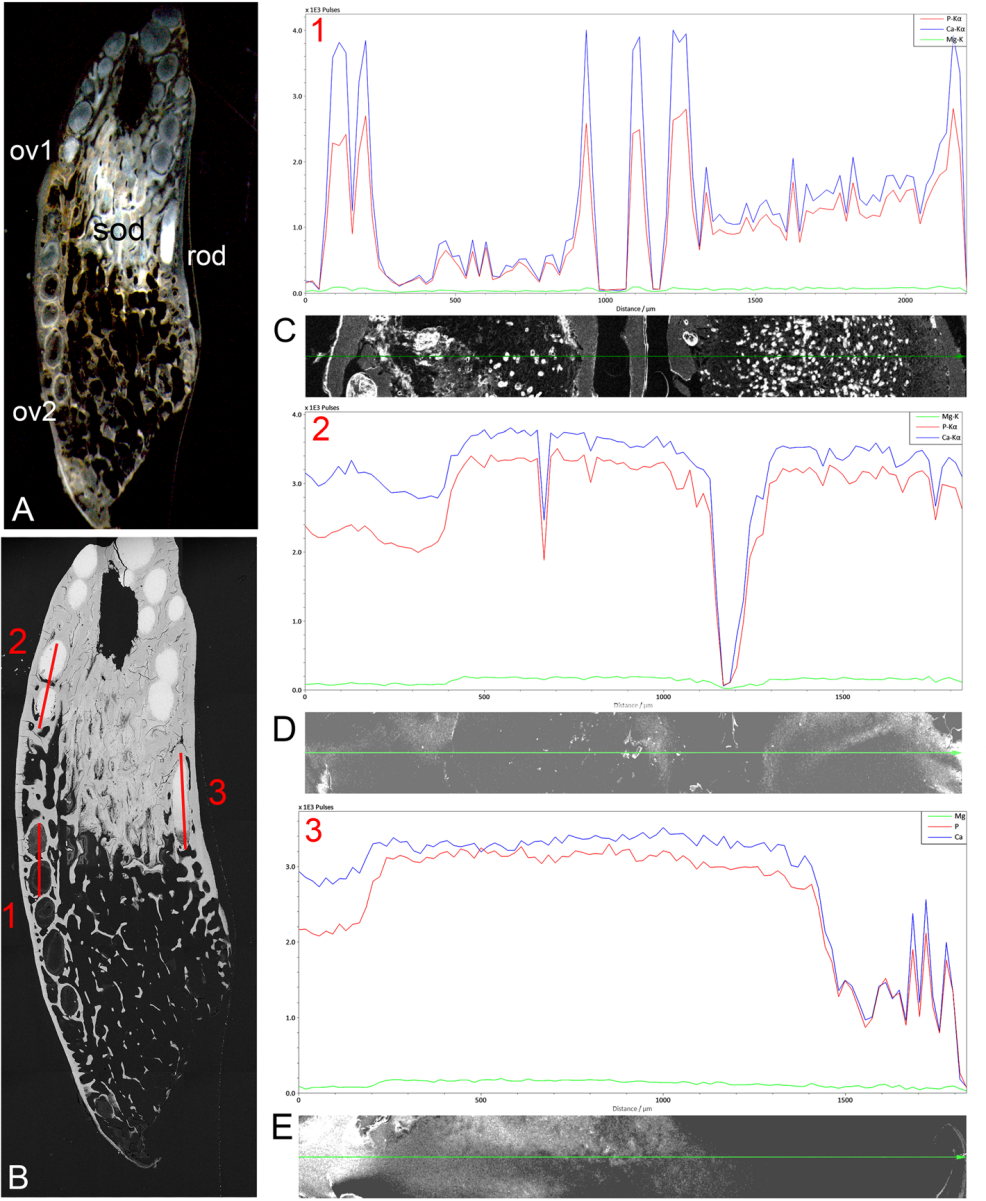
*Harriotta raleighana* (Rhinochimeridae; Holocephali; Chondrichthyes). Magnesium (Mg), Calcium (Ca) and Phosphorus (P) sEDX line plots comparing different regions of upper rostral plate. **a**, **b** reflected light and BSE of labial ovoids, line plots (ov1, 1; ov2, 2) and lingual rod (rod, 3) separated by sclerotic dentine. EDX analyses from aboral to oral, red lines, 1–3 also for tissue types (see Fig. 1a, Fig. 5a, Fig. 7a), × 6.5, × 7. **c** plots 1–3, 1 crosses the empty non-mineralized, two labial ovoids; plot 2 has one ovoid with irregular mineralization (white in A, ov1–1) and incomplete mineralization of second; plot 3 crosses a lingual rod that is fully mineralized orally, with transitional mineralization aborally. Comparing these regions of forming hypermineralized dentine for Ca, P, and Mg values, shows that in plot 1 the trabecular dentine (TD) has higher values than the ovoid space, but itself has two spaces between mineralization peaks, an increase in Mg for each peaks; ov1–2 has begun to mineralize, with Mg up too. Plot 2, Mg increases with increase of Ca and P, higher than trabecular dentine. Ovoids have more mineral than the trabecular dentine with an unmineralized gap between. Plot 3 along the rod, after a slightly lower level aborally, has a continuous high level of mineral, about the same value as in the ovoids, including elevation of Mg. Key to plots, green- Mg; blue- Ca; red- P

**Fig. 15:**
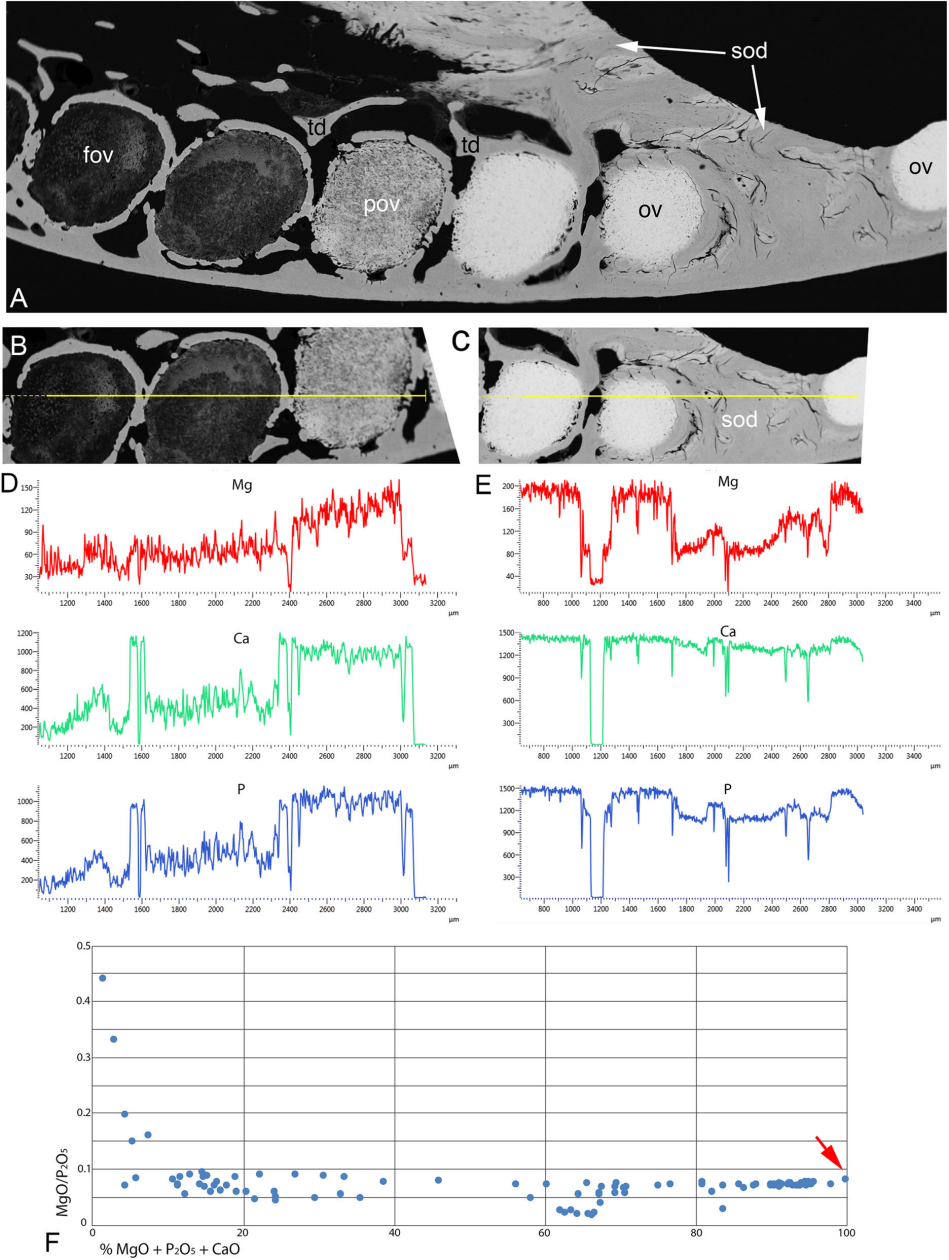
*Harriotta raleighana* (Rhinochimeridae; Holocephali; Chondrichthyes). Magnesium (Mg), Calcium (Ca) and Phosphorus (P) elemental plots of adult upper rostral dental plate of selected areas. **a** SEM of polished surface through labial ovoids close to worn surface of sclerotic dentine (sod); note the increasing degree of mineralization in the ovoids (ov) towards the oral surface (pov, partially mineralized ovoid, fov, forming ovoids). **b** line plot through the barely mineralized, forming ovoids, with distributions of Mg, Ca and P ions changing relative to tissue mineralization, note **d** increase in levels of Mg as well as Ca and P in the partially mineralized ovoid (the elemental plots are not to the same scale). **c** line plot through the hypermineralized ovoids within sclerodentine (sod), with **e** much higher values for all mineral ions of ovoids but Mg relatively less in the sclerotic osteodentine, i.e. distributions of Mg, Ca and P ions changing relative to tissue mineralization. **f** plot of MgO/P_2_O_5_ against total MgO + P_2_O_5_ + CaO for hypermineralized dentine, incorporating data from both ovoids and tritors of lower and upper tooth plates of the same individual; *n* = 110 square area analyses. Pure whitlockite (red arrow) would be expected to have a MgO/P_2_O_5_ ratio of approximately 0.081

From EDX elemental analyses of both upper and lower tooth plates, qualitative line traces confirm that HD in both ovoids and tritors is considerably more densely mineralized than either early trabecular dentine or sclerotic osteodentine (as above, Fig. 12c). Whilst line scans (Figs. 14 and 15a, b and c) provide a qualitative elemental count, point and area scans provide a more quantitative percentage composition. These relate to specific morphological areas of none, or minimal, mineralization, in initial, or transient mineral phases (Fig. 15a, d), within both ovoids and tritors. Many elements were present in negligible quantities, with only Mg, Ca and P being consistently present. Na and Cl were present in some analyses, always in association, and probably represent remnant salt from seawater. In all analyses the total was less than 100%; the remainder of the total is composed of elements that were not detected, presumably C, O and H, as well as pore space.

The fully mineralized HD has a total mineral content (expressed as MgO + CaO + P_2_O_5_) of 95–98%, with less highly mineralized areas showing a full range of mineral density (Fig. 14c, Fig. 15b, d). Not only are mineral densities in the HD and the trabecular dentine distinct (Figs. 12, 13, 14 and Fig. 15a), but elemental analyses also demonstrated a clear mineralogical difference. The MgO/P_2_O_5_ ratio for early trabecular dentine is typically 0.02–0.04 (Fig. 15d), suggesting that some Mg-bearing mineral, probably Mg whitlockite (ß-Ca_3_ Mg_x_(PO4)_2)_) is present, but subordinate to hydroxyapatite. In contrast, The MgO/P_2_O_5_ ratio for HD is typically 0.07–0.08, suggesting dominance of Mg whitlockite. However, some of the least mineralized regions are even more strongly enriched in MgO, equivalent to the opaque in transmitted light region within ovoids described above, suggesting the presence of a different mineral phase. The amorphous structure of these poorly mineralized ovoids and tritors suggests that the earliest phase of mineral deposition was poorly crystalline. Whilst the mineralogy of this material is currently under investigation, hydroxyapatite has the capacity to incorporate a significant quality of Mg when poorly crystalline, unlike when it possesses a well-developed crystalline lattice. It is thus possible that early Mg bearing hydroxyapatite is replaced by Mg whitlockite during the mineralization process. The lowest MgO/P_2_O_5_ ratios within the areas of HD are present in samples at the margins of HD, where it is forming and also bordering trabecular dentine, where the spaces for cell bodies are located (Fig. 4e, f and Fig.6d, e and f).

In contrast, the mineral composition of trabecular dentine was found to be consistent through the dental plate from the growing aboral surface to the worn oral surface, with MgO + CaO + P_2_O_5_ totals of 75–85%, with the remainder composed of OH and HCO_3_ ions from hydroxyapatite and also possibly organic material in cavities and microporosity. Total percentage of the mineral composition, as MgO + CaO + P_2_O_5_ of the later formed sclerotic dentine, is similar to that of the trabecular dentine. No analyses recorded trabecular dentine tissue with very low total mineral percentages; this is probably due to the mineralization of trabecular dentine being continuous within the predentine organic matrix as a distinct mineralization front at each surface within forming dentine, with partially mineralized trabecular dentine never being present. The latter sclerotic dentine typically has a MgO/P_2_O_5_ ratio of 0.03–0.06, higher on average than trabecular dentine, but overlapping with it, suggesting that a larger whitlockite component is often present.

As pure Mg whitlockite would be expected to have a MgO/P_2_O_5_ ratio of 0.081 (Fig. 15f, red arrow), the presence of MgO/P_2_O_5_ ratios close to this and a high MgO + CaO + P_2_O_5_ total would suggest that almost pure whitlockite is present in some HD, with only very small amounts of hydroxyapatite and/or microporosity. Whilst there is no indication of any significant mineral phase other than whitlockite and hydroxyapatite in well mineralized tissue, some analyses of poorly mineralized ovoids reveal very high MgO content, far higher than can be accommodated within whitlockite (Fig. 15f, left side of the graph), also supported by the raised Mg readings relative to the other elements in the least mineralized parts of the plate (Fig. 15b).

### Juvenile and adult dental plates compared

**Fig. 16:**
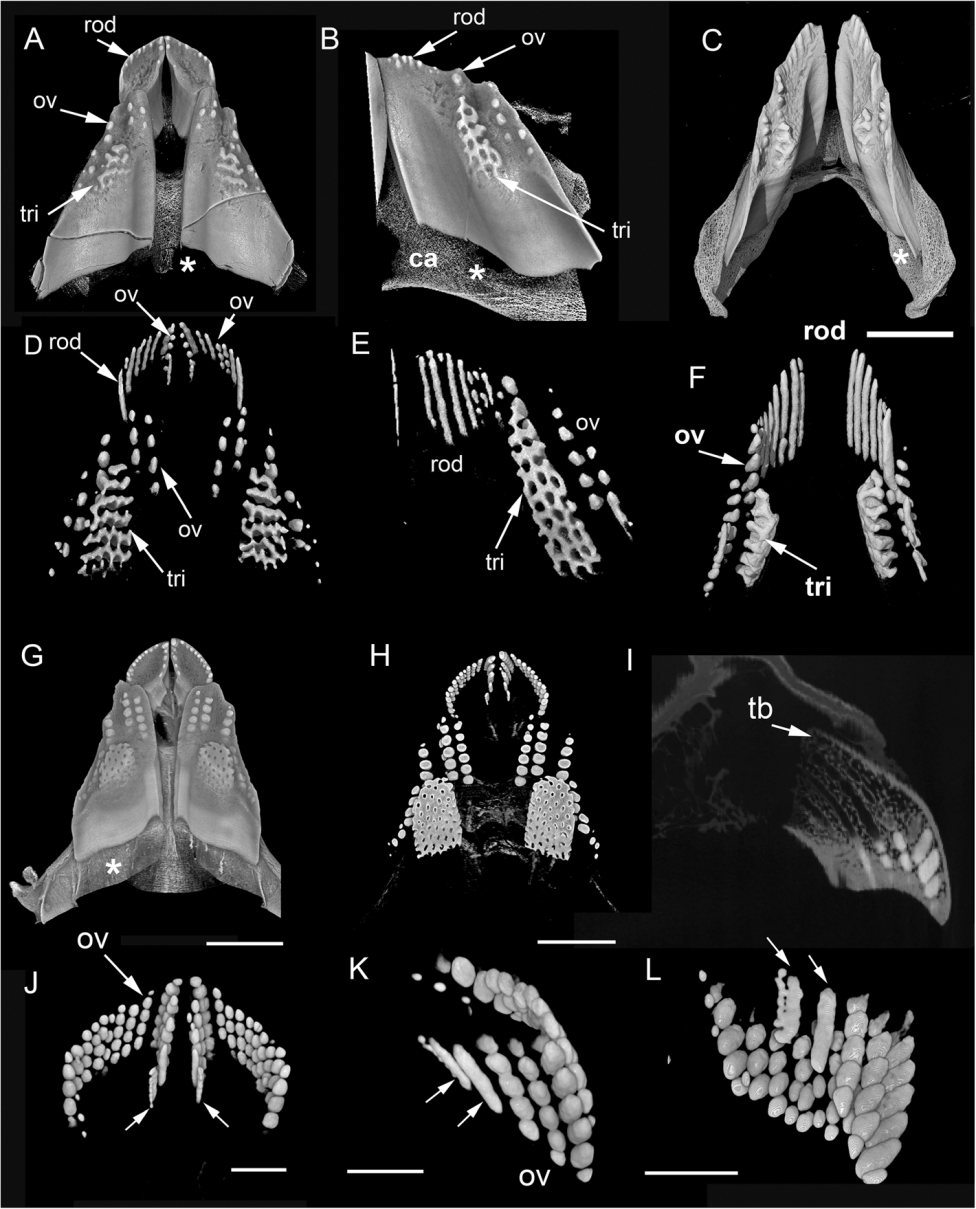
*Harriotta raleighana* (Rhinochimeridae; Holocephali; Chondrichthyes). Juvenile and adult dental plates, μCT-scans density-rendered to expose rods, ovoid and tritors. **a**, **b** juvenile upper and lower dental plates, with rods (rod), ovoids (ov) and developing tritor (tri). **d**, **e** dental plates density-rendered showing (**D**), elongate hypermineralized dentine rods more rostral labially in the upper dental plate, with a row of ovoids and rods parasymphyseally and two series of ovoids among the series of rods; in the more caudal plate, ovoids and a developing tritor are present. **c**, **f** lower jaw from younger individual, indicated by less well-developed tritor and longer rods rostrally. **g**–**l** adult upper dental plates. **h** density-render showing ovoids dominant in rostral plate, ovoids and tritor in more caudal plate. **i** virtual section through rostral dental plate, showing elongate spaces developing within the trabecular dentine (td). **j**–**l** density-rendered rostral plate in **j** oral, **k** symphysial, **l** symphysial (rotated) view. Small white arrows indicate rods of dentine developing caudally. **l** Most caudal rod appears paired, possibly due to failure of spaces in trabecular dentine to form properly. Asterisk (A–C, G) indicates jaw furrow in which dental plate develops. Scale bar C = 0 .5cm, G, I = 1 cm, J–L = 0.25 cm

As noted in the section ‘Adult *Harriotta raleighana* tissue morphology’, although dental plates from a juvenile individual of *Harriotta raleighana* have HD in both tritors and ovoids located within the supporting structure of the trabecular dentine (Fig. 1c and Fig. 16a, b, c, d, e and f), they appear to be morphologically different at the oral surface. As in adults, juvenile dental plates are renewed at the aboral surface where capsules of trabecular dentine (see ‘Ovoids as hypermineralized dentine HD’) define unmineralized spaces, clearly shaped as rods, ovoids and tritors (Fig. 13b,c, d, e, g, arrow heads). Although mineralized ovoids and tritors develop as in the adult plates, in the juvenile upper rostral and lower dental plate multiple rods are seen rostrolingually (Fig. 16b, c, e, f). In virtual section (Fig. 14a, b and c) these rods form via mineralization that reaches the same density level as adult HD (Fig. 14e, scan 3). Also in the adult upper rostral plates (Fig. 16g, h, i, j,k and l), rod-shaped HD is present symphyseally and caudally within the plate (Fig. 16j, k and l), and develops similarly to the rods in the juvenile dental plate, by infilling an elongated, preformed space in the trabecular dentine (Fig. 16i). The rods form at the caudal end of a row of more typical ovoids (Fig. 16j, k, white arrows), with the last rod being misshapen, as if a larger and smaller rod had fused at multiple points (Fig. 16l) rather than developing separately.

### Summary of adult dental plate structure

**Fig. 17:**
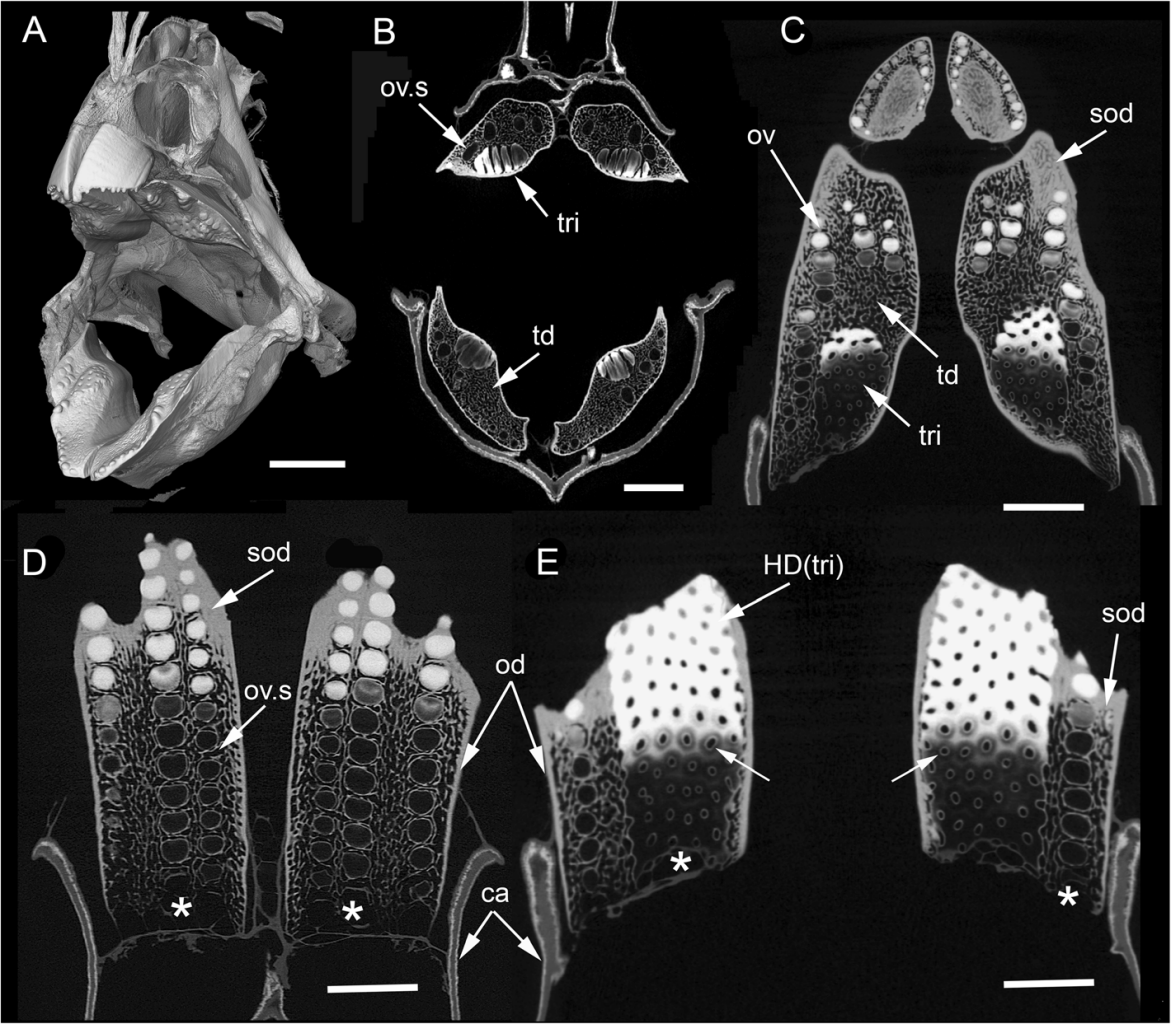
*Harriotta raleighana* (Rhinochimeridae; Holocephali; Chondrichthyes). Adult μCT adult jaws with dental plates rendered in situ with virtual sections. **a** skull in rostrolateral view showing upper and lower dental plates. **b** virtual frontal section, through cartilages of upper and lower jaws, showing positions of hypermineralized dentine (eg., tritor, tri) within trabecular dentine (td), along with developing spaces for tritor and ovoids (eg., ov.s). **c** upper dental plates (rostral and caudal pairs) in a horizontal plane showing developing regions of ovoids (ov) and worn oral surface and tritors (tri) within trabecular dentine. **d** lower jaw, both plates in oblique frontal section, with ovoids only, through rostro-dorsal part showing development of new ovoid spaces within the trabecular dentine (asterisk) and hypermineralisation towards the oral, biting surface. **e** oblique frontal section through more caudal region of lower dental plates, new ovoids along with tritor developing within the trabecular dentine (asterisk), with circumvascular dentine late to mineralize (white arrows), and mineralization towards the oral surface (HD(tri)). Scale bar A = 1 cm, B, C = 0 .5cm; D, E = 0.25 cm

**Fig. 18:**
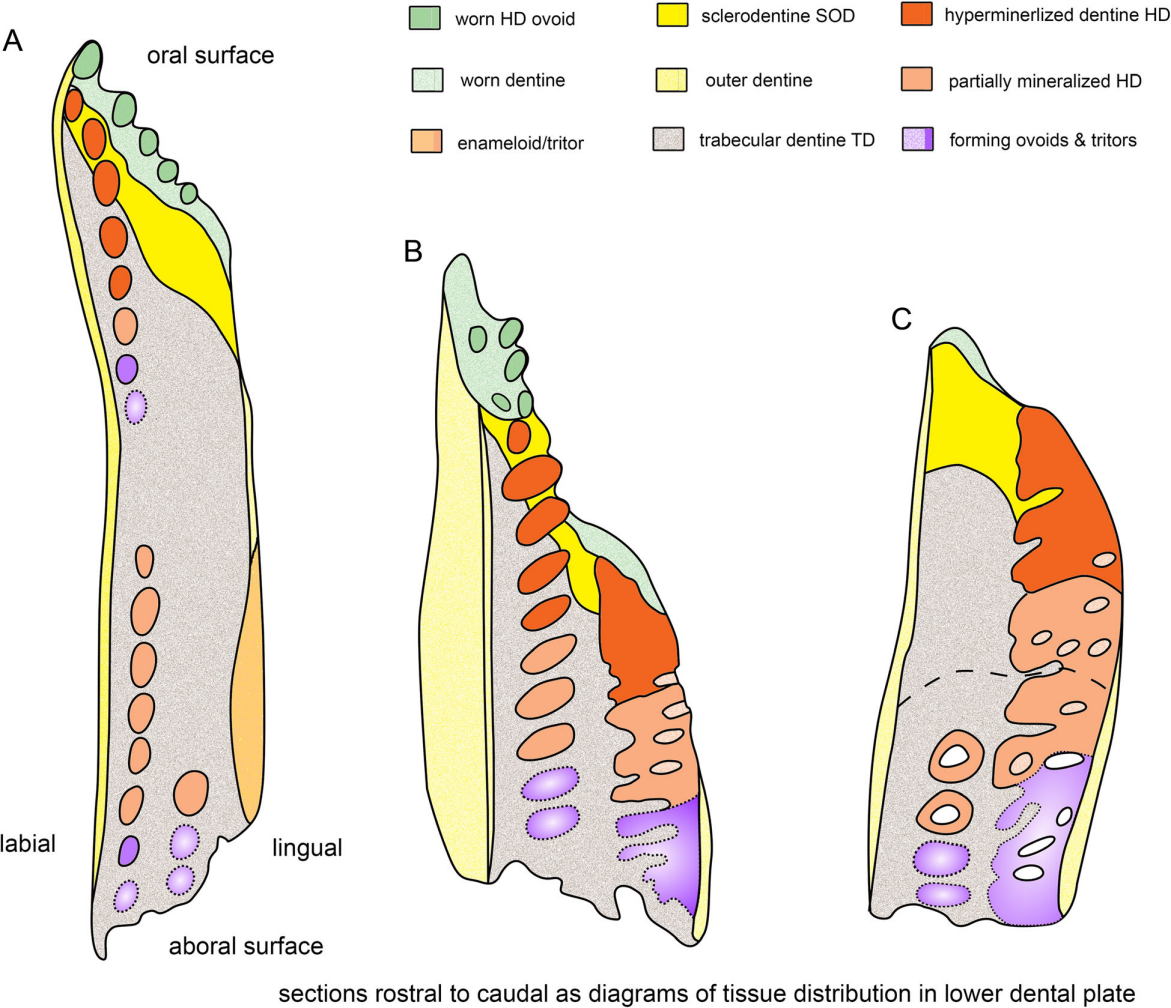
*Harriotta raleighana* (Rhinochimeridae; Holocephali; Chondrichthyes). Summary diagrams representing distribution of all tissue types in a series from rostral to caudal. Lower dental plate as three regions of developing and mature tissues that are colour coded as in the key. **a** rostralmost hard tissue section (Fig. 1a), outer lingual hypermineralized outer layer shown, maybe enameloid, or part of tritor (Fig. 5), **b** mid region from virtual section, worn surface both ovoids and tritor depicted in green (Fig. 2d), **c** caudal most hard tissue section in which a disjunct line shows separation of functional oral tritor from developing aboral tritor and series of new labial ovoids (Fig. 7)

An adult specimen with all six plates in situ (Fig. 17a, b, c, d and e) has worn HD at each oral surface in upper and lower jaws apposed to each other in vivo (Fig. 17b). The ovoids occupy a relatively smaller area than that of the trabecular dentine (Fig. 17b, d, e), and in the upper jaw rostral plate there are only ovoids, whereas ovoids are paired with tritors in the caudal plate (Fig. 17c). All plates are located in poorly mineralized jaw cartilages but both labial and lingual outer dentine is as well mineralized as the trabecular dentine, similar to the sclerotic osteodentine (Fig. 17c, d and e, sod, od, ca). The latter is extensive in this plane (near oral surface of the horizontal section) of the anterior upper plate. The μCT densities are not sufficiently resolved to say how mineralized they are but the tissues are very compact and a similar density to the circumvascular canal infills of the tritors. Many preformed ovoid spaces are present as part of the trabecular dentine, aligned as stacks below the mineralized ones (Fig. 17d, ov.s). Later mineralizing vascular pulpal canals are seen at the mineralizing front of dentine matrix in the forming tritors (Fig. 17e, arrows). The organization of ovoids is mostly in the rostral part of the plate, but also alongside the tritors labially in the lower plate (Fig. 1b, c, d and e, Fig. 17c). The tissue distribution of each type of dentine is colour coded and summarized in Fig. 18 (see in next section ‘Distribution of hypermineralized dentine tissues’).

## Discussion

### Distribution of hypermineralized dentine tissues

*Harriotta* adult dental plates are small compared to the size of the head, and equipped for benthic feeding with multiple functions. The overall dental plate morphology suggesting that they could be used for both gripping and crushing, but without discrete teeth at any stage of development so far observed. We have demonstrated how the tissues are arranged and details of their unique development and mineralogy; previously their anatomy and histology had only been briefly outlined [1, 4, 13]*.* Instead of teeth, hypermineralized dentine (HD, Fig. 18, orange) forms as discrete but differently shaped masses, including rods, ovoids and tritors (Fig. 1d, Fig. 16), each embedded in the less mineralized trabecular dentine during the constant growth of the plate from the aboral surface (Fig. 1a, Fig. 2d). These differences in dentine hardness and distribution contribute to an uneven, worn, oral surface in the adult, with a different shape in the juvenile dentition (Fig. 1b, c; see ‘Tritoral tissue’). Although the term ‘rod’ has been used to describe the more elongate series of ovoids (‘pearlstrings, string of beads’ [1, 4]), we have identified continuous rods of dentine in juvenile dentition and in the adult upper dentition (Fig. 16), and so restrict the term to describe this morphology.

The oral trabecular dentine is progressively mineralized and quantitatively, relative to hydroxyapatite on grey scale density, is half that of the HD (Fig. 12b). Deep to the surface it has been transformed into osteodentine that becomes translucent, and harder as sclerotic osteodentine (Fig. 1a, Fig. 2a, b, sod, Fig. 18, yellow). The labial outer dentine is also well mineralized (as is the lingual outer dentine) and supports the ovoids as a raised edge of the dental plate (Fig. 1a, od, Fig. 18, orange), as well as providing support for the lingual tritors, with both ovoids and tritors probably used for triturating (comminution). The different ways in which tissues mineralize is discussed in ‘Mineralogical changes during HD formation’.

The variation in distribution of each tissue along the rostro-caudal and labio-lingual axes is summarized in diagrams taken from serial, hard tissue sections of a lower dental plate, and virtual μCT sections (Fig. 18). Dental plates form continuously to renew the oral surface (Fig. 18, green) with ovoids arising only within the oval shapes made of trabecular dentine at the aboral surface (Fig. 18, purple), and tritors forming around vascular canals, but enclosed within the trabecular dentine (Fig. 2d, e). Ovoids are aligned in a vertical (oral to aboral) series on the rostro-labial parts of the tooth plate so, like the tritors, we can distinguish immature tissue from mature (Fig. 18, purple to blue to orange) as they are in constant renewal, and given their regular spacing and organization in series within the dental plate, they may be programmed for successive, replacement growth, (see ‘Continuous renewal and replacement by serial successive growth’).

Caudally and lingually, immature tritoral tissue occurs as closely spaced parallel tubes of the trabecular dentine (Fig. 2d, e) containing blood vessels and, we suggest, a lining of mesenchymal pericytes that are potential odontogenic stem cells [14, 15]. Ovoids are shaped by circumferentially arranged capsular fibres as part of the trabecular dentine (Fig. 2c, e), in both arrangements the spaces created by the trabecular dentine are filled with the rapidly mineralizing matrix for the HD (Fig. 2d, e), only later becoming more and more mineralized relative to the trabecular dentine (Figs. 11, 12, 13, 14, 15 and 18, from purple to blue, then orange, see ‘Mineralogical changes during HD formation’).

We cannot determine whether this increase in mineralization of trabecular dentine at the oral surface is prophylactic (forms in advance of wear), or is a cell-directed infilling to form sclerodentine induced by wear as is suggested for pleromic dentine of numerous forms of agnathan dermal armour, or crushing teeth [13]. The level of mineralization in the HD of tritors and ovoids may also be influenced by the degree of wear. The elemental data for both suggests that the whitlockite-rich dentine (HD), is developmentally late relative to the whitlockite-poor trabecular dentine forming the trabecular framework of the whole plate that supports the localized HD. This framework itself is becoming more compact (osteodentine), and hardened with the addition of more mineral into sclerodentine, a term we propose for trabecular dentine as it becomes more mineralized towards the oral surface (Fig. 12a, b, Fig. 15a, Fig. 18, yellow). As with the increase in mineralization, it is currently unknown whether the development of these Mg-rich materials is simply developmentally late (details in ‘Mineralogical changes during HD formation’), forming after the trabecular dentine framework, and is independent of wear, or controlled by wear as a biological feedback mechanism.

### Continuous renewal and replacement by serial successive growth

The distribution of HD (ovoids, rods, tritors) in the dental plate is regulated by the formation of unmineralized spaces within the trabecular dentine, as a part of renewal at the aboral surface to make the characteristic pattern for the species. In particular this distribution is shaped by vascular canals in the trabecular dentine that organize the tritors, and vascular capsules that supply the ovoids (Fig. 2d, e). This is growth related to renewal because the dental plates are continuously growing (statodont) and said not to be lost and then replaced [11]. New concepts, describe a residual mesenchymal cell population, perhaps pericytes as mentioned earlier (‘Distribution of hypermineralized dentine tissues’), that can be triggered into forming odontoblasts (dentine-depositing cells), from studies of the continuously growing incisors in mice, could be applied to this renewal type of growth [14, 15].

However, as described above, we also have data for iterative growth as replacement (not renewal), in serial hard tissue sections through the adult plate, where we observed a disjunct surface running through the plate caudally, with changing levels of mineralization and altered structure on either side of this surface (Fig. 7a, b and c, Fig. 8a, b, Fig. 18c, dashed line). We suggest that this indicates a form of replacement growth, in which a second more aboral tritor was forming, within the outer dentine, in the caudal part of the dental plate, abutting the functional and still mineralizing tritor. This, together with an aboral series of forming ovoids labially, but absent in the oral region above (Fig. 7d), shows that the ovoids are new, and we would suggest co-ordinated, with the new tritor below the disjunct surface. Thus, these ovoids, as well as the tritor, are interpreted as part of an iterative succession in a replacement series that will become more mineralized before becoming functional at the oral surface. Other evidence is provided by a more rostral section through the same plate (Fig. 1a, ov1, ov2), where two series of ovoids are well separated by trabecular dentine, hence may form a replacement series in the region where there is no tritor.

We were able to test these observations made in the lower dental plate by examining serial ground sections of the upper caudal (posterior) dental plate of the same individual in incident, reflected light, and with BSE, where a similar disjunction separated the oral from the aboral parts of the tritoral HD (Fig. 8a, b and c, arrows). In these examples from both upper and lower dentitions, we found the same disjunction in backscattered electron (BSE) images, confirming a common replacement event, but because BSE imaging is only from a shallow depth, this line was less distinct (Fig. 8c, d and e, arrows). Nevertheless, it is clear from BSE mode that in the lingual tritor, a junction is present between much less mineralized aboral HD and more mineralized oral HD, supporting results from incident light (Fig. 8a, b, black, white arrows).

These observations can be best explained as the aboral development and incorporation of a new tritor block and stack of ovoids, developing from interaction between pulpal tissue and an omnipresent outer dental epithelium (details in ‘Cells involved in production of dentine types’), comparable to the dental lamina associated with tooth development in sharks. This continues to demarcate the area of tooth plate aborally [5] and production of all tooth plate tissues is generated here, to a distinct repetitive pattern. With respect to the latter, pre-odontoblast stem cells (see below ‘Cells involved in production of dentine types’) associated with trabecular dentine development, and the specifically located spaces for rods, ovoids and tritors, may be the regulators of this timed tissue production, induced by mesenchymal cells in contact with vascular endothelium [14, 15]. Therefore, although teeth per se, are absent from the dental plate in chimaeroids such as *Harriotta*, they may be represented by these proposed mechanisms for replacement that are comparable with those characteristic of elasmobranchs as well as stem-group holocephalans.

### Cells involved in production of dentine types

The tissue for ovoids and tritors is formed from a different type of odontoblast than those forming the trabecular dentine, as they are linked in a row lining the forming dentine surface and each occupying a large cell body space (Fig. 3b, c, Fig. 6, cbs). Massive numbers of tubules ramify into the mineralizing tissue, linking all mineralized tissue to blood vessels (Fig. 4). We suggest these cells differentiated from perivascular stem cells, comparable to the mouse model where perivascular niches have mesenchymal stem cells (pericytes) that can differentiate into specialized tooth mesenchyme-derived cells, or odontoblasts during tooth growth that makes new dentine [16]. Ishiyama et al. [17] also proposed a specialized cell for the HD, but coming from an osteoblast lineage, however, there is a complete absence of bone tissue in these dental plates (i.e. no osteoblasts). Bone is absent in extant chondrichthyans, and only rarely present in fossil sharks [12].

Specialized HD secreting cells in *Harriotta* are also distinguished by the enormous number of tubules that radiate throughout the mineralizing tissue of ovoids and tritors (Fig. 2b, c, Fig. 3b, c, Fig. 4e, f, Fig. 6a, b, c, d, e and f) and in scanning electron microscopy of the section surfaces (Fig. 8d, e and f, Fig. 9e, f and g, Fig. 10a, b, c, d, e, f, g, h and i) form the complex network that is transformed into vesicles and saccules. The first mineralized granules of the matrix are found in some of these membranous vesicles, and these observations concur with Ishiyama et al. [17] who described similar features from semithin sections, without convincingly showing the cells. Nevertheless, they also described tubular saccules, derived from tubules of those cells specifically involved in the early mineralization of this HD in holocephalans (see §2.4). Ishiyama et al. [17] described these cells as pleromoblasts and adopted the name pleromin for the tissue (see §2.3; [1, 4, 5, 13]). We propose, that new names are used, whitlockin and whitloblasts, as so many of the details in formation and structure are different from that of pleromin [5], especially the incorporation of magnesium (see ‘Mineralogical changes during HD formation’).

In the trabecular dentine, the relationship of cells to matrix is very different as cell processes bend and branch to ramify in a network through the tissue in a chaotic arrangement, but also with odontoblasts that are adjacent to vascular capillaries (Fig. 4a, b, c, and d). However, the outermost dentine layer has cell body spaces external to the trabecular dentine, with tubules running into the inner layer of trabecular dentine (Fig. 4a, b, c and d, od, cbs, id). This direction of formation suggests a most unusual event where odontogenic stem cells have migrated from the pulpal tissue to the outer surface of the forming dental plate.

Late closure of the vascular spaces in the trabecular dentine occurs from the activity of the same odontoblasts, by forming the circumvascular fibres that transform trabecular dentine into osteodentine (Fig. 4a, os). Then extra mineralization orally hardens this osteodentine into sclerotic osteodentine, forming a more compact tissue with denteones, analogous with osteones in compact bone (Fig. 2a, os). However, this sclerodentine is not hypermineralized, with levels of Ca density that are about half that of the HD (Fig. 12d). Impregnation of this dentine with mineral towards the functional surface is suggested to also be under control of these odontoblast cells (see **‘**Adult *Harriotta raleighana* tissue morphology’; and ‘Mineralogical changes during HD formation’).

### Mineralogical changes during HD formation

Initial mineralization on the aboral surface of the dental plates forms a loose and open framework of trabecular dentine, including rod, ovoid and tritor spaces. This material appears mineralogically homogeneous and uniform (Figs. 14 and 15) with elemental analysis indicating a mixture of hydroxyapatite and a minor component of Mg-bearing whitlockite [6]. The mineral density and composition of the framework trabecular dentine does not vary appreciably between the aboral region and the worn oral surface, suggesting that there is no recognisable mineralogical change once this framework dentine has been mineralized. In the sclerotic osteodentine, where all vascular spaces have been infilled and the whole tissue is more mineral dense (Fig. 12a, b), MgO content is, on average, higher than that of the trabecular dentine alone, although there is very considerable overlap of the fields. Thus this later more mineralized tissue appears to be relatively richer in Mg whitlockite, although hydroxyapatite is still the dominant biomineral. By comparison, mineralization increases rapidly in ovoids and tritors, so that up to six individual ovoids may simultaneously show early stages of mineralization, changing suddenly from poorly mineralized to fully mineralized HD (Fig. 11a, b and c).

We found that MgO/P_2_O_5_ ratios suggest that almost pure Mg whitlockite is present in the HD of highly mineralized ovoids and tritors, with only trace amounts of hydroxyapatite (Fig. 9g, Fig. 11e). Very early on in the mineralization sequence, Mg is extremely enriched, far more so than in whitlockite. This Mg-rich mineral phase is very poorly crystalline and potentially marks the initiation of mineral growth. There is no evidence of a mineral other than those recorded elsewhere being present, and it is possible that poorly crystalline hydroxyapatite forms the host of this additional Mg, with the Mg being ejected from the hydroxyapatite lattice as the degree of crystallization increases. Raised MgO/P_2_O_5_ levels in partially mineralized ovoids and tritors (above that of whitlockite) suggest a mixing trend of this early mineral phase that itself is combined with larger quantities of Mg whitlockite and Mg bearing hydroxyapatite. The low total Mg, even where it is proportionally enriched (Fig. 15b), indicates that further Mg is added during later phases of mineralization, and the Mg is not simply transferred from this early mineral phase to whitlockite. There is thus no evidence for remobilisation and reprecipitation of dentine material, which is only possible via osteoclastic remodelling available to all jawed vertebrates, except chondrichthyans [18].

Whitlockite contains only about 20% of Mg-Fe-containing beta tricalcium phosphate [19], and in this study Fe + was absent from the mineral phase. Whitlockite has been recognized in vertebrate mineralized tissues [20]; as reviewed by Laskus and Kolmus [21], Indeed, Mg is the fourth most common element in the human body, and as much as 60% deposited in bone. Chondrichthyans lack bone, with whitlockite being first recognized in holocephalans in the extant *Chimera phantasma* [6]. Biological whitlockite contains acid phosphate groups (HPO_4_^2−^), and tends to be stable at lower pH, for example, research on human enamel with calculus formation shows that whitlockite is the most stable calcium phosphate below pH 5.5 [19]. In dental calculus whitlockite is 2% *w*/w, or 90% of the calculated value of Ca_20_Mg(PO_4_)_14_ (for details see [19]), but the mineral is generally so variable that details of its composition and properties are not possible to define. Related to their important biological role, particular attention has focused on tricalciumphosphates (TCP) and ‘additives’ such as magnesium ions, all of which have been widely investigated as non-apatitic CaPs substituted with various ions for medical reconstruction of mineralizing organic matrices [21]. The presence of whitlockite may thus have selective physiological advantages but studies of this are still in early stages.

Concerning the possible organic matrix within the dental plate, Ishiyama et al. [6] observed via TEM (transmitted electron microscopy) a matrix of scattered, sparse reticulin, many and various membranous vesicles, some myelinated. Our observations with Nomarsky optics and BSE (Fig. 9g, h) complement theirs, showing dominant tubules and saccules in early stages of HD tissue (pleromin), from cells they named pleromoblasts. We also found little evidence of organized matrix fibres, with the only linear organization of mineral aligned along the tubule walls within otherwise disorganized mineral (Fig. 9c, d). There is little birefringence (i.e. not organized into collagen bundles) of the early matrix but when mineralized there is weak birefringence in both tritor and ovoid, deduced as from small mineral crystals at right angles to each other in an otherwise disorganized matrix (Fig. 11d, e [14]). In experimental production of whitlockite the crystals are so small that they preclude detection by polarized light [19].

The HD tissue of the *Harriotta* dental plates needs a new name, because we suggest that pleromin, although it is commonly used in the literature, is unsuitable. Because of its hardness and translucency, and deposition into a pre-existing mineralized framework, it conforms to the definition of pleromic, or infilling, dentine (‘true pleromic dentine’ [22, 23]), as defined in many other composite plated dentitions [9, 13, for discussion and earlier references; 6, 14]. However, because the early stages of HD involve deposition of granular crystals, and a transformation phase that may be quite different from known biological methods of making a matrix-free hypermineralized tissue without epithelial cell involvement like enameloids, we suggest a change in name. Also this tissue is composed of a different form of calcium phosphate especially rich in magnesium (whitlockite [6, 17]), rather than crystals of hydroxyapatite as in all other mineralized connective dental tissue, and could be given a new name specific to holocephalans, like ‘petrodentine’ for dipnoan tooth plates [23]. We suggest **‘whitlockin’** for holocephalan hypermineralized dentine as a more specific term, replacing pleromin, and for the specialized cells **‘whitloblasts’** instead of pleromoblasts (see §2.3).

### Enameloid tissue

One enigma is the statement that enameloid is not found in holocephalans, but is present in all teeth of selachimorphs (sharks and rays), as well as on their dermal placoid scales and some Palaeozoic sharks (reviewed in [24]). However, we have observed a hypermineralized outer layer in sections, but only at the most rostrolingual surface of the dental plate, where the tritor is not present (Figs. 5 and 18). The layer is above a trabecular dentine support, but is at the surface and as highly mineralized as the lingual tritor that forms internally within the trabecular dentine framework. Judging from the position and angle of incremental lines it was possibly mineralized from as yet unknown outer layer cells. Also on the surfaces of whole dental plates, incremental lines can be seen with difficulty, all support layering from the outside, but none of this data is definitive for enameloid being present in *Harriotta*. However, the direction of deposition, together with a patchy +ve blue colour as the sign of birefringence when the layer is at + 45° to the polars (Fig. 5d), is probably due to partial alignment of mineral crystals that are normal to the surface, but the origin of the tissue is equivocal. All examinations seem to show lines, but they are far from certain in surface view, as this layer only exits on the most rostro-symphysial aspect of the lower tooth plate. This is a topic for further research, as clearly more data is required to decide if this layer is enameloid with an epithelial cell input, or thin, extended tritoral dentine, as the presence or absence of tubules is as equivocal, as is the direction of formation.

### Juvenile and adult dentitions

Juvenile and adult dental plates include the rods, tritors and ovoids developing initially within preformed spaces within the trabecular dentine. Ovoids and rods are positioned rostrolabially, with tritors on the caudal or lingual side of the dental plate. The rods, more characteristic of the juvenile dentition, are replaced by ovoids in the adult dentition except in the rostral dental plate of the upper jaw where they are present caudally, located within a row of ovoids along the parasymphyseal margin of the plate. Two individuals show differing surface structure of the tritoral region, with density dissections and virtual slices suggesting they are at different growth stages. In the putatively older specimen, the tritors are larger and better developed (Fig. 14a, b and , versus Fig. 13c, f, g, h), including large foramina for canals. In the younger specimen, the tritors are smaller and not fused, and vascular canals are not completely infilled on the oral surface.

In addition, in the older specimen the rostral rods are shorter, while in the younger specimen, these appear longer. We suggest that in the older specimen, the process of rod development has ceased, with the rods removed at the oral surface with wear. This may mark a transition to the adult stage, and would involve a reset of the developmental program rostrally, from spaces for continuous rods to those for separate ovoids. This changes how the trabecular dentine could preform the space for the HD, with patterning that maintained the labio-lingual spacing of the rods but converted that for the rods into oro-aboral series of ovoids. Rods are retained in the upper adult dental plate, with two rods being present most caudally and fused (Fig. 15k, l), suggesting that the developmental reset has not occurred here. In shark dentitions, later-developing teeth closer to the jaw joint are often smaller and more rudimentary than earlier teeth e.g. [25], as if the morphogenetic programme is reduced, something analogous could result in fusion of caudal rods in *Harriotta* adults.

## Conclusions

A key to understanding how the statodont dentition of *Harriotta* functions without teeth is the arrangement of the hypermineralized dentine within the dental plate. This hypermineralized tissue forms initially as a Mg-rich, amorphous phase transforming into granules, later being replaced by Mg whitlockite (MTCP, ß-Ca_3_ Mg_x_(PO4)_2_), the production of which continues into late mineralization. The proposed name for this mineralized tissue is ‘whitlockin’ rather than pleromin, providing a dentition that is unique because of this significant whitlockite forming dense mineralized tissue within a trabecular dentine  dominated by hydroxyapatite. As well, mineralization occurs within distinctive, fragmented parts of the dominating, extensive cell processes, with only scant organic matrix. This hypermineralized dentine in the separately organized ovoids and tritors ensures an oral surface of projecting hard elements arranged to a specific pattern, within less hard dentine (Figs. 15 and 16a, c, d; green, Fig. 18).

The statodont dentition with six composite dental plates (Figs. 15 and 16a) is renewed by constant growth aborally of trabecular dentine, framing the initial ovoids and tritors within a shell of outer dentine. Unusually this tissue is made by odontoblasts from the outer surface apposing the inner dentine (pale yellow, Fig. 18). The pattern and shape of ovoids and tritors is preformed by the trabecular dentine, with a notable vascular system for the tritors, comprising vascular pulpal tissues and localized stem cells. The latter is a resource to renew odontoblasts as well as the larger, specialized cells that secrete the whitlockin (whitloblasts, as opposed to pleromoblasts; Figs. 1 and 17d, e, asterisk; Fig. 18, purple, see, ‘Cells involved in production of dentine types’). Mineralization occurs rapidly, first as irregular groups of granules located within a maze of whitloblast tubules, these becoming a mass of saccules and membranous bodies.

Early mineralization reveals a very high MgO content, far higher than can be accommodated within whitlockite (Fig. 15f) and likely to be part of an amorphous hydroxyapatite phase. This is supported by elevated Mg readings relative to the other elements in these least mineralized parts of the dental plate (Fig. 15d) and likely represents Mg-rich and poorly crystalline hydroxyapatite. This is present very early on in the mineralization sequence, a novel observation that correlates with the lacunae of optically dense, non-birefringent, minimal mineral density material in the same early mineralizing ovoid (Fig. 11), a non-crystalline phase.

As well as regular continuous renewal aborally, new evidence from caudal sections of the dental plates shows an abrupt histological change in the mineralizing tritors, interpreted as disjunct development within the plate. We propose this as evidence for a mechanism for serial replacement of individual tritors, that represents a mode of tooth replacement, potentially comparable to that present in elasmobranchs, and in extinct holocephalan taxa.

Many of these features are novel, presenting a unique dentition among chondrichthyans, including new modes of dentine deposition and mineralization, that may prove to be general for rhinochimaeroid holocephalans (whitlockite being also present in *Chimaera*), and perhaps the Holocephali more broadly.
